# BIRC6 mediates imatinib resistance independently of Mcl-1

**DOI:** 10.1371/journal.pone.0177871

**Published:** 2017-05-16

**Authors:** Denis O. Okumu, Michael P. East, Merlin Levine, Laura E. Herring, Raymond Zhang, Thomas S. K. Gilbert, David W. Litchfield, Yanping Zhang, Lee M. Graves

**Affiliations:** 1 Department of Pharmacology, School of Medicine, University of North Carolina, Chapel Hill, North Carolina, United States of America; 2 Lineberger Comprehensive Cancer Center, University of North Carolina, Chapel Hill, North Carolina, United States of America; 3 Department of Chemistry, University of North Carolina, Chapel Hill, North Carolina, United States of America; 4 UNC Michael Hooker Proteomics Center, University of North Carolina, Chapel Hill, North Carolina, United States of America; 5 Department of Biochemistry, Schulich School of Medicine and Dentistry, Western University, London, Ontario, Canada; 6 Department of Radiation Oncology, University of North Carolina, Chapel Hill, North Carolina, United States of America; Universita degli Studi di Firenze, ITALY

## Abstract

Baculoviral IAP repeat containing 6 (BIRC6) is a member of the inhibitors of apoptosis proteins (IAPs), a family of functionally and structurally related proteins that inhibit apoptosis. BIRC6 has been implicated in drug resistance in several different human cancers, however mechanisms regulating BIRC6 have not been extensively explored. Our phosphoproteomic analysis of an imatinib-resistant chronic myelogenous leukemia (CML) cell line (MYL-R) identified increased amounts of a BIRC6 peptide phosphorylated at S480, S482, and S486 compared to imatinib-sensitive CML cells (MYL). Thus we investigated the role of BIRC6 in mediating imatinib resistance and compared it to the well-characterized anti-apoptotic protein, Mcl-1. Both BIRC6 and Mcl-1 were elevated in MYL-R compared to MYL cells. Lentiviral shRNA knockdown of BIRC6 in MYL-R cells increased imatinib-stimulated caspase activation and resulted in a ~20-25-fold increase in imatinib sensitivity, without affecting Mcl-1. Treating MYL-R cells with CDK9 inhibitors decreased BIRC6 mRNA, but not BIRC6 protein levels. By contrast, while CDK9 inhibitors reduced Mcl-1 mRNA and protein, they did not affect imatinib sensitivity. Since the Src family kinase Lyn is highly expressed and active in MYL-R cells, we tested the effects of Lyn inhibition on BIRC6 and Mcl-1. RNAi-mediated knockdown or inhibition of Lyn (dasatinib/ponatinib) reduced BIRC6 protein stability and increased caspase activation. Inhibition of Lyn also increased formation of an N-terminal BIRC6 fragment in parallel with reduced amount of the BIRC6 phosphopeptide, suggesting that Lyn may regulate BIRC6 phosphorylation and stability. In summary, our data show that BIRC6 stability is dependent on Lyn, and that BIRC6 mediates imatinib sensitivity independently of Mcl-1 or CDK9. Hence, BIRC6 may be a novel target for the treatment of drug-resistant CML where Mcl-1 or CDK9 inhibitors have failed.

## Introduction

Chronic myelogenous leukemia (CML) is a malignancy of myeloid cells characterized by accumulation of mostly myeloid cells in the bone marrow and bloodstream [1,2]. CML is a result of the fusion of the breakpoint cluster region (Bcr) and Abelson (Abl) genes due to reciprocal translocations between chromosomes 9 and 22, t(9;22), resulting in a chimeric, constitutively active Bcr-Abl tyrosine kinase [1,3–9]. While successfully treated with the Bcr-Abl kinase inhibitor imatinib, high incidences of disease relapse and drug resistance have been recorded in CML patients [4,5,8,10,11].

Imatinib mesylate (IM, Gleevec^®^, STI571, CGP57148B), the first clinically available kinase inhibitor, is an ATP-competitive inhibitor of Bcr-Abl developed as a frontline treatment for CML [4,12,13]. Some of the IM therapy-related (Bcr-Abl-dependent) mechanisms of resistance include Bcr-Abl amplification or expression of inhibitor-resistant Bcr-Abl with mutations in the kinase domain. For example, the T315I “gatekeeper mutation” diminishes the kinase’s affinity for the drug. Additional evidence suggest that imatinib resistance is due to Bcr-Abl independent mechanisms like enhanced expression of drug exporters (like P-glycoprotein) or activation of alternative kinase signaling cascades [7,14,15]. These challenges have led to the development of second generation (dasatinib and bosutinib) and third generation (ponatinib) inhibitors that target both Bcr-Abl and Src family kinases [8,12,16]. Alternatively, hematopoietic stem cell transplantation remains the only other feasible cure for refractory CML. Unfortunately, most patients cannot benefit from this approach due to advanced age at diagnosis or lack of a suitable stem cell donor [3,17].

Inhibitors of apoptosis proteins (IAPs) are a group of highly evolutionarily conserved anti-apoptotic proteins known to globally regulate caspases and immune signaling [18–23]. Studies have shown that up-regulation of IAPs such as cellular inhibitor of apoptosis protein 2 (cIAP2), X-linked inhibitor of apoptosis protein (XIAP), survivin and others correlates with decreased apoptosis and increased drug resistance [20,24–28]. Non-IAP anti-apoptotic proteins of the Bcl-2 family such as myeloid cell leukemia-1 protein (Mcl-1), are known to also mediate drug resistance in diverse cancers [15,29,30]. Mcl-1 is a well-known marker of anti-apoptosis whose role in cancer drug resistance is well documented [31–34]. It is localized to the outer mitochondrial membrane where it heterodimerizes with and neutralizes pro-apoptotic Bcl-2 proteins like Bak, Bim, Noxa, and PUMA resulting in suppression of cytochrome c release and prevention of apoptosis [14,15,29,31–34]. We previously observed that Mcl-1 is increased in the imatinib-resistant CML cell line (MYL-R) characterized by overexpression and activity of the Src family kinase, Lyn [14,29,35,36]. These results suggest that Mcl-1 may play a role in the Bcr-Abl-independent, Lyn-mediated imatinib resistance in these cells [14,15,29]. This is consistent with several studies over the last decade that have focused on Mcl-1 as a key anti-apoptotic protein mediating drug resistance in various human cancers [30,33,34,37–39]. The development of effective Mcl-1 inhibitors, however, has met various challenges with the few commercially available candidates having limited success in the clinic [38–41].

Baculoviral inhibitor of apoptosis repeat-containing protein 6 (BIRC6), also known as Apollon or BRUCE, is a member of the BIR domain containing family of IAPs [20,22,28,42]. BIRC6 is localized to the trans-Golgi membrane and vesicular networks, and possesses a single BIR domain of 75 amino acid residues arranged in tandem repeats in the N-terminal region of the protein. This domain is the region that binds and inhibits caspases, thereby preventing caspase activation required for apoptosis [20]. BIRC6 has a UBC domain at its C-terminus that allows it to function as a chimeric E2/E3 ubiquitin ligase, one target being the pro-apoptotic protein, Diablo [20,43,44]. This domain is also a binding site for mitochondrial proteins like Diablo/Smac and HtrA2/Omi that antagonize BIRC6’s anti-apoptotic activity in cells. It was shown that BIRC6 only binds and inhibits caspases as a homodimer, and that caspases could cleave this dimer and degrade BIRC6, thereby quenching its anti-apoptotic properties [20]. Thus, the reciprocal inhibition between BIRC6 and caspase activation is a potential mechanism by which cells may coordinate survival and apoptotic processes [20,43,45,46].

BIRC6 has been shown to be important in cell cycle progression and DNA damage repair where it functions as a scaffold protein for assembly of the DNA damage repair machinery [47–49]. Furthermore, several *in vivo* studies show that total ablation of BIRC6 causes growth retardation and, embryonic and perinatal lethality, underscoring the protein’s critical role in cell viability [43,50,51]. Recent studies show that BIRC6 is increased in a number of intractable human cancers, including *de novo* acute myeloid leukemia, breast cancer, ovarian cancer, hepatic cancer, prostate cancer, colon cancer, neuroblastoma, and non-small-cell lung cancer where it may contribute to cancer cell survival and proliferation [19,21,23,45,52–55]. Despite increased interest in BIRC6 and its role in cancer cell survival, its regulation and involvement in mediating drug resistance is not well understood.

In this study, we examined BIRC6 regulation in an imatinib-resistant cell line (MYL-R) and compared this to its imatinib-sensitive counterpart (MYL). MYL-R cells are independent of Bcr-Abl mutations, amplification or overexpression of multi-drug resistance proteins like P-glycoprotein [15,56]. Our studies show that BIRC6 expression is increased in this cell line, and that BIRC6 knockdown is sufficient to restore imatinib sensitivity. Our data further show that Lyn activity is important for up-regulation of both Mcl-1 and BIRC6 in MYL-R cells, and suggest that BIRC6 plays a dominant role in mediating imatinib resistance in these cells. Lastly, our results suggest that Lyn-dependent BIRC6 phosphorylation may regulate BIRC6 stability by preventing degradation by caspases. In summary, these studies suggest BIRC6 may be a promising target for the treatment of some drug resistant human cancers.

## Materials and methods

### Cells, cell culture and reagents

MYL and MYL-R human CML cell lines were generous gifts from Dr. Hideo Tanaka (Department of Haematology and Oncology, Hiroshima University, Hiroshima, Japan) [15]. K562 cells, another CML cell line, were bought from American Type Culture Collection (ATCC) (Manassas, VA). Cells were cultured in culture flasks suspended in RPMI 1640 medium (Gibco^®^ by Life Technologies^™^, U.S.A.) supplemented with 10% fetal bovine serum (Atlanta Biologicals; Norcross, GA), and 1% antibiotic/antimycotic (Invitrogen; Carlsbad, Ca). Cells were maintained at 37°C in a humidified 5% CO_2_ atmosphere in concentrations of approximately 0.6x10^6^ cells mL^-1^. Culture medium was replaced every 2 to 4 days. For most experiments described here, cells were harvested by low-speed centrifugation and washed with 1X PBS prior to lysis.

Reagents were obtained from the following sources: ponatinib and dasatinib were from LC Laboratories (Woburn, MA); Z-VAD-FMK, imatinib, dinaciclib, and flavopiridol were from Selleckchem (Houston, TX); and HY-16462 was from MedChem Express (Monmouth Junction, NJ). The primary human antibodies used include: BIRC6 (Abcam, Cambridge, MA and Cell Signaling Technology, CST, Danvers, MA), Cytochrome c (Abcam), CDK9, phospho-CDK9, phospho-Src (Y416), PARP/Cleaved PARP, phospho-c-Abl (CST), phospho-EEF1D, phospho-CK2beta, phospho-IF2B (Litchfield Lab, University of Western Ontario, Canada), Lyn, Mcl-1, c-Abl, alpha-Tubulin, Hsp60, Erk2, and β-actin (SCBT); with secondary antibodies, anti-mouse and anti-rabbit IgG-HRP conjugated (Promega {Madison, WI}). Phospho-EEF1D, phospho-CK2beta, and phospho-IF2B antibodies were diluted per supplier recommendations: 1:20,000, 1:10,000, and 1:10,000 respectively in 3% bovine serum albumin (BSA) in Tris-buffered saline supplemented with Tween-20, TBS-T (10 mM Tris-HCl pH 7.6, 150 mM NaCl, 0.05% Tween-20). All other primary antibodies were diluted following supplier recommendations: 1:1000 in 5% BSA/TBS-T. Secondary antibodies were diluted at 1:10,000 in 5% dry, non-fat milk in TBS-T.

### Multiplexed inhibitor bead (MIB) affinity chromatography / MS analysis

Kinases were isolated from MYL and MYL-R cell lysates as previously described [14,57]. Briefly, cells were harvested by centrifugation and washed once with PBS. Cells were lysed in MIB Lysis Buffer [50 mM HEPES pH 7.5, 150 mM NaCl, 0.5% Triton X-100, 1 mM EDTA, 1 mM EGTA, 10 mM NaF, and 2.5 mM Na_3_VO_4_, supplemented with protease inhibitor cocktail (Roche) and phosphatase inhibitor cocktails 2 & 3 (Sigma-Aldrich)]. Lysates were sonicated, clarified by centrifugation, and filtered through a 0.2 μm syringe filter. The amount of starting material was 5 mg protein, and was diluted to 1.25 mg/mL with MIB lysis buffer. Diluted lysates were passed over a mixture of 117uL each of the following kinase inhibitors conjugated to ECH Sepharose beads: Purvalanol B, VI-16832, and PP58, layered from top to bottom respectively. The kinase inhibitor-bead conjugates were previously equilibrated in high salt buffer (50 mM HEPES pH 7.5, 1 M NaCl, 0.5% Triton X-100, 1 mM EDTA, and 1 mM EGTA). MIBs columns were sequentially washed with high salt buffer, low salt buffer (50 mM HEPES pH 7.5, 150 mM NaCl, 0.5% Triton X-100, 1 mM EDTA, and 1 mM EGTA), and SDS buffer (50 mM HEPES pH 7.5, 150 mM NaCl, 0.5% Triton X-100, 1 mM EDTA, 1 mM EGTA, and 0.1% SDS). Proteins were eluted by boiling samples in elution buffer (100 mM Tris-HCl pH 6.8, 0.5% SDS, and 1% B-mercaptoethanol) for 15 minutes twice. Dithiothreitol (DTT) was added to a final concentration of 5 mM and samples were incubated at 60°C for 25 minutes. Samples were then cooled to room temperature on ice and alkylated by adding iodoacetamide to a final concentration of 20 mM and incubating for 30 minutes in the dark at room temperature. Samples were then concentrated in 10K Amicon Ultra centrifugal concentrators (Millipore) followed by methanol and chloroform precipitation of proteins. The final protein pellets were re-suspended in 50 mM HEPES pH 8.0 and incubated with trypsin at 37°C overnight. Residual detergent was removed by three sequential ethyl acetate extractions then desalted using Pierce C-18 spin columns (Thermo Scientific) according to the manufacturers protocol. Samples were run on the Q-Exactive HF mass spectrometer (see LC/MS/MS analysis). Kinases were quantified by label-free analysis of kinase peptides using the MAXQUANT software package with integrated search engine (ANDROMEDA). Peptides required a minimum length of six amino acids and protein identification required at least two unique peptides. The cutoff of global false discovery rate for peptides and proteins was set at 1% and only unique peptides were used for label free quantification.

### Trypsin digestion and phosphopeptide enrichment

For phosphoproteome analysis, 4 volumes of cold acetone were added to 1 mg of each lysate for protein precipitation. The samples were then reconstituted in 7 M urea, reduced, alkylated, and digested overnight with trypsin (Promega). Peptides were desalted using Sep-Pak C18 cartridges (Waters) according to manufacturer’s protocol, then dried down and stored at -80°C until further use. Phosphopeptide enrichment was performed using the 200 μl TiO_2_ Spin Columns from GL Sciences. Peptide samples were reconstituted with 80/20 ACN/lactic acid in 1% TFA, then loaded onto the TiO_2_ spin column prewashed with 80% ACN, 1% TFA. Peptides were washed once with 80/20 ACN/lactic acid in 1% TFA and twice with 80% ACN, 1% TFA. Retained peptides were eluted twice with 20% ACN, 5% NH_4_OH and acidified < pH 4 with formic acid. All phosphopeptide eluates were desalted using C18 Spin Columns (Thermo Fisher) then dried down and stored at -80°C until further use.

#### LC/MS/MS analysis

Each sample was analyzed by LC-MS/MS using an Easy nLC 1000 coupled to a QExactive HF equipped with an Easy Spray source (Thermo Scientific). First, samples were reconstituted in loading buffer (1% ACN, 0.1% formic acid), and then loaded onto a PepMap 100 C18 column (75 μm id × 25 cm, 2 μm particle size) (Thermo Scientific). Peptides were separated over a gradient consisting of 5–32% mobile phase B over 60 min at a 250 nl/min flow rate, where mobile phase A was 0.1% formic acid in water and mobile phase B consisted of 0.1% formic acid in ACN. The QExactive HF was operated in data-dependent mode where the 15 most intense precursors were selected for subsequent fragmentation. Resolution for the precursor scan (m/z 400–1600) was set to 120,000 with a target value of 3 × 10^6^ ions. For MS/MS scans with HCD (normalized collision energy 27%), resolution was set to 15,000 with a target value of 2 × 10^4^ ions. Peptide match was set to preferred, and precursors with unknown charge or a charge state of 1 and >7 were excluded.

### Data analysis

Raw data files were processed using MaxQuant software (version 1.5.3.17). Data were searched against a human UniProt database (downloaded Aug 2015) using the integrated Andromeda search engine. The following parameters were used to identify tryptic peptides for protein identification: up to two missed trypsin cleavage sites; carbamidomethylation (C) was set as a fixed modification; and oxidation (M), deamidation (NQ), and phosphorylation (STY) were set as variable modifications. A false discovery rate (FDR) of 1% was used to filter all results, and match between runs was enabled. Bioinformatics analyses were performed with Perseus software (version 1.5.3.0). Phosphorylation sites with a localization probability of at least 0.70 were considered.

### Immunoblot analysis

Cells were harvested and lysed in a modified RIPA (RIPA, no SDS) buffer (150 mM NaCl, 9.1 mM Na_2_HPO_4_, 1.7 mM NaH_2_PO_4_, 1% NP-40, and 0.5% deoxycholic acid; adjusted to pH 7.4) and supplemented with 2 mM sodium orthovanadate, 10 mM NaF, 0.0125 μM calyculin A, and cOmplete Protease Inhibitor Cocktail (Roche Diagnostics, U.S.A.). The lysates were clarified by centrifugation and the protein concentrations were normalized using a Bradford assay (with reagents from BIO-RAD). Samples for gel electrophoresis were prepared by adding protein lysates to Laemmli sample buffer (final concentration: 0.25 M Tris pH 6.8, 10% glycerol, 5% β-mercaptoethanol, 0.001 μg/mL Bromophenol blue) and 30 μg of protein were loaded into each well of an SDS-polyacrylamide gel for protein separations. Proteins were transferred to polyvinylidene difluoride (PVDF) membranes (BIO-RAD) which were then blocked for 1 hr with 5% non-fat dry milk or 5% BSA dissolved in Tris-buffered saline supplemented with Tween-20 (TBS-T). The membranes were then incubated in primary antibodies at 4°C overnight, washed 3 times with TBST, then incubated with anti-mouse / rabbit IgG-HRP conjugated secondary antibodies for 1 hr at room temperature. The membranes were rinsed 3 times with TBST then developed using Clarity^™^ ECL Western Substrate (BIO-RAD), and imaged using a ChemiDoc^™^ Touch Imaging System (BIO-RAD).

### RNA extraction and cDNA synthesis

Total RNA was extracted and purified using RNeasy^®^ Mini Kit (Qiagen, U.S.A.) according to the manufacturer’s protocol. cDNA was synthesized from reverse transcription on 1.5 μg total RNA in a 50 μL reaction using High Capacity cDNA Reverse Transcription Kit (Applied Biosystems, U.S.A.) and iCycler (BIO-RAD, U.S.A.), according to the manufacturer’s protocol.

### Quantitative real-time PCR (qRT-PCR)

The cDNA was analyzed by real-time qPCR using TaqMan^™^ Gene Expression Assays Kit and TaqMan^™^ 2X Universal PCR Master Mix (Applied Biosystems by Life Technologies) on an Applied Biosystems 7500 Fast Real-Time PCR System. All procedures followed company protocol.

### Cell viability assay

Cell viability was determined by seeding triplicate populations of MYL-R or MYL or BIRC6 knockdown MYL-R cells on a 96-well plate at 5x10^3^ cells/well in 100 μL culture medium supplemented with various concentrations of kinase inhibitors. Cells were incubated at 37°C / 5% CO_2_ for 72 hours. 20 μL of Resazurin^™^ (SIGMA) MTS assay reagent was added to each well, and the plate returned to the incubator for ~ 2 hrs. Cells were transferred into opaque, black 96-well assay plates (Costar) and fluorescence measured at 520 nm using the PHERAstar microplate reader Spectra Max Plus 384 (BMG Labtech). Cell viability was assessed using GraphPad Prism SoftWare version 6.05 (GraphPad Prism, Inc). The data presented for each cell viability assay is representative of three independent experiments performed for each cell line.

### shRNA knockdown of BIRC6

pLKO.1 lentiviral vectors containing shRNA directed against BIRC6 (TRCN0000004157, 58, 59, 60, and 61) or a non-targeting shRNA (shCtrl) were purchased from the UNC Lenti-shRNA Core Facility. Lentivirus transduction of MYL-R cells with shRNA was done per the protocol supplied by the RNAi Consortium (http://www.broadinstitute.org/rnai/public/resources/protocols). Briefly, MYL-R cells were seeded at 5x10^5^ cells/mL in 5 mL growth media containing 8 μg/mL polybrene, and incubated with 1 mL of viral shRNA overnight. Stably transduced cells were selected for by exposure to 2 μg/mL puromycin in cell culture [14]. The cells were harvested one week after selection and immunoblot analysis performed to determine which shRNA strain was most effective in knocking down BIRC6.

### shRNA knockdown of Lyn

pLKO.1 lentiviral vectors containing shRNA directed against Lyn (TRCN0000010101, 04, 05, 06, and and 07) or a non-targeting shRNA (shCtrl) were purchased from the UNC Lenti-shRNA Core Facility. Lentivirus transduction of MYL-R cells with shRNA was done per the protocol supplied by the RNAi Consortium, and as outlined above for BIRC6. One week after puromycin selection, BIRC6 mRNA and protein were analyzed by QRT-PCR and immunoblot respectively.

### shRNA knockdown of CDK9

shRNA directed against CDK9 (TRCN 0000000494, 495, 496, 497, and 498) or a non-targeting shRNA (shCtrl) were purchased from Thermo Scientific and packaged into pLKO.1 lentiviral vectors by the UNC Vector Core as described above (for BIRC6 and Lyn). Lentivirus transduction of MYL-R cells with shRNA was done per the protocol supplied by the RNAi Consortium. The transiently transduced MYL-R cells were harvested at 48 hrs post-infection and BIRC6 mRNA and protein were analyzed by qRT-PCR and immunoblot. Transient transduction was used in order to avoid substantial loss in cell viability previously observed upon knockdown of the protein and selection with puromycin.

### Caspase-3/7 activity assay

BIRC6 protein was knocked down in MYL-R cells with shRNA transcripts shBIRC6-58 and shBIRC6-59. A third population of the same MYL-R cells was transduced with shCtrl. After selection and stabilization of the transduced cells with puromycin, 3x10^6^ cells from each population were treated with 1 μM imatinib (Selleckchem) and a DMSO control, with an additional treatment of parental MYL-R cells with a 1 nM dasatinib (control). After 24 hours, cells were collected from each flask, lysed on ice for 10 minutes with 300 μL lysis buffer [50 mM HEPES (pH 7.4), 5 mM CHAPS, and 5 mM DTT], and lysates clarified by centrifugation at 10,000xg for 10 minutes at 4°C. Protein concentrations were normalized using the Bradford Assay. In a 96-well plate, 100 μg of protein was added to 200 μL of assay buffer [20 mM HEPES (pH 7.4), 0.1% CHAPS, 2 mM EDTA, 5 mM DTT, and 15 μM Ac-DEVD-AMC caspase-3/7 substrate (Sigma-Aldrich)] in triplicate, and the plate incubated at room temperature in the dark for 2 hours. The fluorescence intensity from liberated AMC (7-amido-4-methylcoumarin) was measured using a PHERAstar microplate reader (BMG Labtech, Cary, NC) with 360 nm excitation and 460 nm emission filters.

### Detection of apoptosis by flow cytometry

Apoptotic cells were detected using the simultaneous dual mitochondrial membrane potential detection and caspase activity assay kit, MitoCasp^™^ (Cell Technology), according to the manufacturer’s protocol. MitoCasp^™^ is a cell permeable two-color stain used to simultaneously detect caspase activity and mitochondrial membrane potential (DeltaPsi, ΔΨm) using flow cytometry. For detection of caspase activity, MitoCasp^™^ uses cell permeable, non-toxic carboxyfluorescein (FAM) labeled fluoromethyl ketone (FMK)-peptide inhibitors of caspases that covalently bind to the active caspases thereby allowing for analyses by flow cytometry of cells containing bound inhibitors. For ΔΨm detection, MitoCasp^™^ uses a cell permeable, low toxicity, and low ΔΨm independent (non specific) binding cationic dye that has a strong fluorescent signal in the red region. Healthy cells accumulate the dye in their mitochondria in proportion to the ΔΨm, mostly resulting in high fluorescence intensity. Apoptotic cells have compromised ΔΨm that prevents the dye from accumulating in the mitochondria thereby yielding low fluorescence signals.

Briefly, three separate populations of approximately 6x10^5^ cells/mL BIRC6 knockdown MYL-R cells (shCtrl, shBIRC6-58, and shBIRC6-59) were treated as described in caspase-3/7 activity assay above. After 24 hours of apoptosis induction, 300 μL of cells from each cell line was transferred to a 5 mL FACS tube prior to staining for flow cytometry analysis as described below. Appropriate controls were included as necessary. Approximately 6x10^5^ cells/mL of Lyn knockdown MYL-R cells (shCtrl, shLyn-01, shLyn-04, shLyn-05, shLyn-06, and shLyn-07) were cultured overnight. The cells were prepared for flow cytometry analysis as described above for BIRC6 knockdown cells, with appropriate controls.

#### Flow cytometry

Cells were stained with 10 μL each of the 30X ΔΨm dye and 30X Caspase detection reagent. Single-stain controls were included for instrument compensation and background correction. Cells were gently vortexed and incubated for 60 min at 37°C/5% CO_2_. They were washed twice with 2 mL of 1X Wash Buffer, and then resuspended in 300 μL of 1X Wash Buffer before filtering with 30 μm filters (Becton Dickinson, BD, Biosciences). The cells were quickly analyzed by measuring fluorescence using the FL1 (FITC filter) channel (for caspase detection reagent) and FL2 (Phycoerytherin:PE filter) channel (for ΔΨm dye) of a fluorescence-activated BD Fortessa flow cytometer (Excitation: 488 nm). At least 10,000 cells were determined for each sample. The flow cytometry data were analyzed by FlowJo v10 software.

The percent apoptotic cells represent the fraction of cells in Q3 defined by a loss in mitochondrial membrane potential and gain in caspase activity (S4 Fig).

### Detection of Cytochrome c release

Cytochrome c release was measured using Cytochrome c Releasing Apoptosis Assay Kit (Abcam, U.S.A.), per the manufacturer’s protocol. Briefly, approximately 1x10^8^ MYL-R cells were treated with 5 nM ponatinib or DMSO for 24 hours. Cells were harvested and washed with 10 mL cold 1X PBS. Each cell population was split into two and one pair of cell pellets were lysed in immunoblot lysis buffer and protein concentrations normalized using the Bradford assay. Pellets from the second set were resuspended in 1.0 ml of the supplied 1X Cytosol Extraction Buffer Mix containing DTT and protease inhibitors (Abcam, USA). After incubating the cells on ice for 10 minutes, they were homogenized in an ice-cold Dounce tissue grinder (Wheaton, USA) as outlined in the protocol (Abcam, USA). This process was repeated with fresh 1.0 ml of 1X Cytosol Extraction Buffer Mix due to the large number of cells. Aliquots of the crude lysates were saved before centrifuging the lysates at 10,000xg for 30 min at 4°C and collecting the supernatant as Cytosolic Fractions. The pellets were resuspended by vortexing for 10 seconds in 0.2-ml Mitochondrial Extraction Buffer Mix containing DTT and protease inhibitors (Abcam, USA) and saved as Mitochondrial Fractions. Protein concentrations for the crude lysates, Cytosolic Fractions, and Mitochondrial Fractions were normalized using the Bradford assay and appropriate amounts of 4X Reducing Sample Buffer added. Immunoblot analyses were performed on all lysates.

### Statistics

Data are reported as the mean ± standard error of the mean (S.E.M); S.E.M. was performed on all datasets to determine positive and negative error. Two-tailed student t-test was used to make comparisons between groups, and *p* values below 0.05 at the 95% confidence level were considered to be statistically significant. Calculations were performed using GraphPad Prism and Microsoft Excel.

## Results

### BIRC6 mRNA and protein are increased in imatinib-resistant MYL-R cells

**Fig 1:**
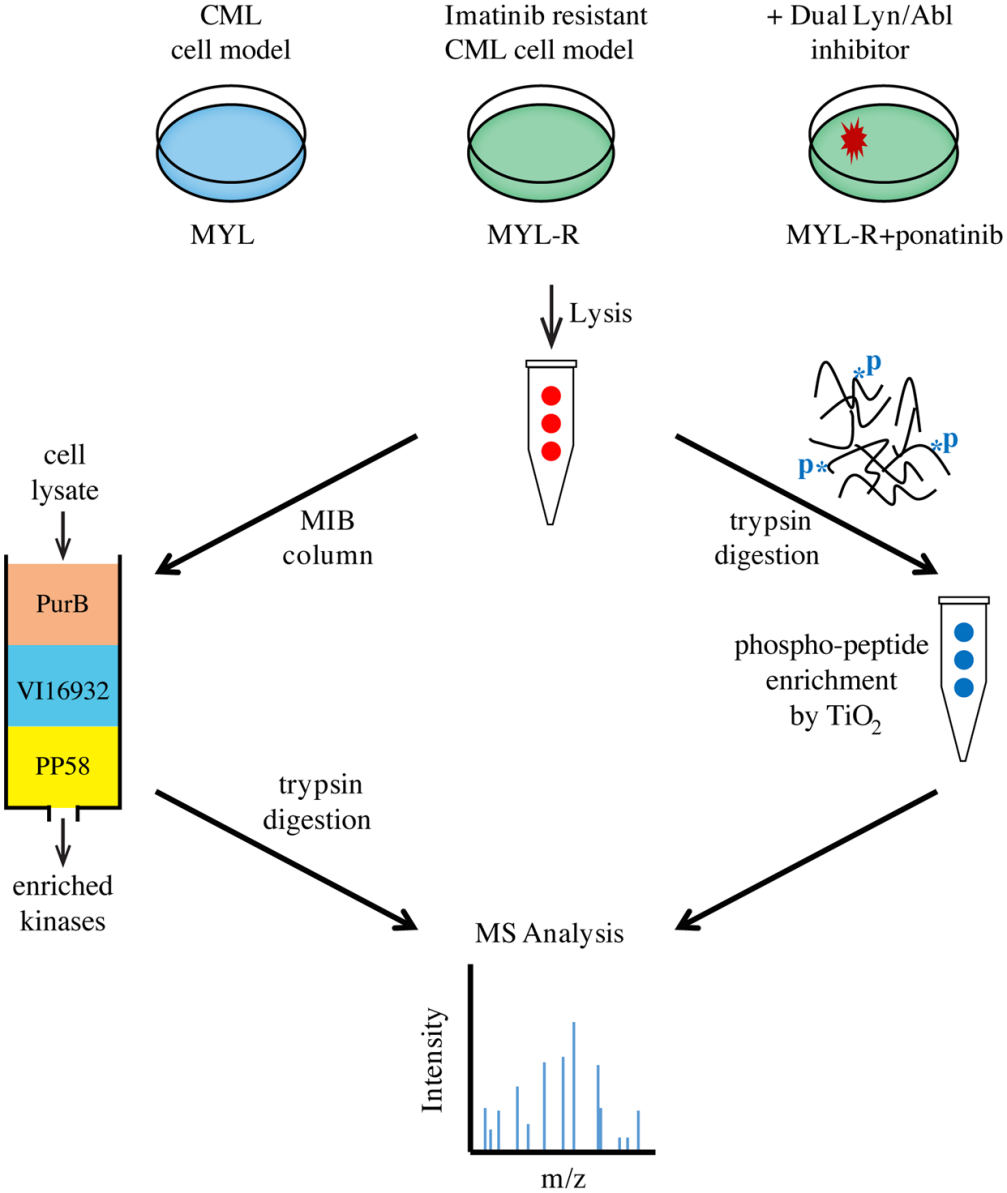
Combined MIB/MS and phosphopeptide enrichment strategy for studying proteome dynamics in CML cells. MIB/MS was used to study kinome dynamics in MYL, MYL-R, and MYL-R cells treated with ponatinib (10 nM, 1 hr.). In parallel, phosphoproteomics was used to study global phosphorylation differences from the same cells. Identification of peptides was accomplished by LC-MS/MS and label-free quantification (LFQ) of mass spectral data was performed using MaxQuant and the integrated ANDROMEDA search engine [61].

**Fig 2:**
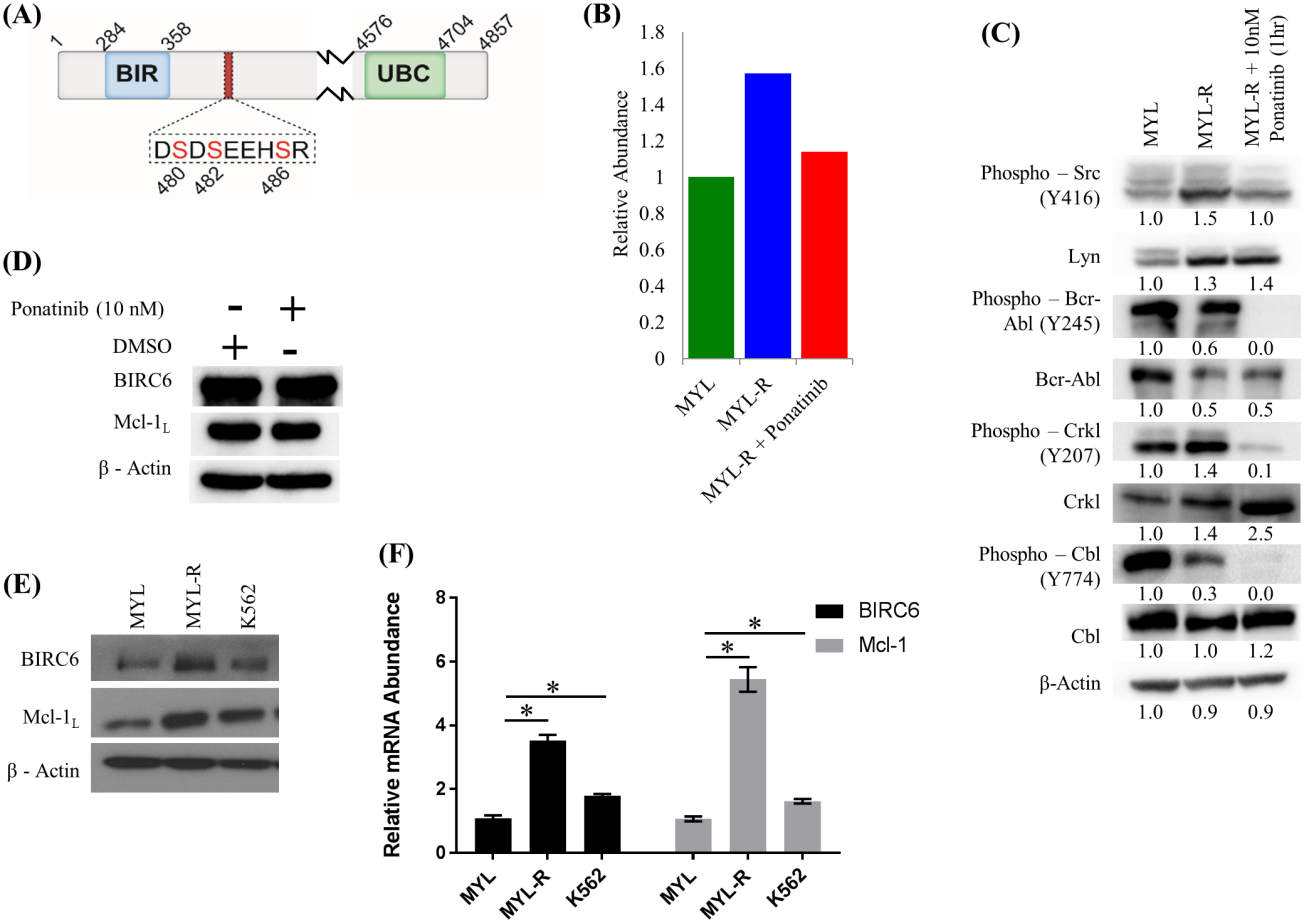
BIRC6 mRNA, protein, and BIRC6 phosphopeptide are higher in imatinib resistant MYL-R cells. (A) Sequence and position of the BIRC6 phosphopeptide. (B) Short-term ponatinib treatment of MYL-R cells reduced BIRC6 phosphopeptide. The BIRC6 phosphopeptide was isolated from cell lysates of MYL, MYL-R, and MYL-R cells treated with 10 nM ponatinib or 0.1% DMSO for 1 hour. Label-free quantification of mass spectral data was done using MaxQuant and normalized to MYL. (C and D) Short-term ponatinib treatment suppressed Bcr-Abl and Lyn signaling in MYL-R cells. Total BIRC6 and Mcl-1 proteins were not affected. Immunoblot analyses of the same lysates were performed to validate changes in Bcr-Abl and Lyn observed from the MIB/MS data. (E and F) BIRC6 mRNA and protein were elevated in MYL-R cells compared to MYL and K562 cells. QRT-PCR and immunoblot analyses were performed as described in Materials and Methods on parental MYL, MYL-R, and K562 CML cell lines to examine BIRC6 expression. * Represents *p < 0*.*05*. Data are representative of three independent experiments.

In a previous study we compared the functional kinomes of MYL and MYL-R cells [14] using MIB/MS technology [57] and showed that Lyn was substantially up-regulated in MYL-R compared to MYL cells [14]. Other studies showed that elevated Lyn activity mediated imatinib resistance in these cells [15,58]. Hence, we further examined both the functional kinome and phosphoproteome of these cells to identify phosphorylation events that may contribute to imatinib resistance (Fig 1). MYL-R cells were treated for 1 hour with 10 nM ponatinib, a dual Bcr-Abl and Lyn inhibitor [12,16], or DMSO and MIB/MS kinome profiling and phosphopeptide enrichment were performed as described in Materials and Methods. This concentration of ponatinib was sufficient to potently inhibit cell viability (S1 Fig). Untreated MYL cells were also included. The MIB/MS technology can be used to assess inhibitor specificity [59]. With short-term (1 hour) treatment, kinases bound by ponatinib will no longer be available to bind to inhibitor beads, and therefore show a decrease in MIBs capture. The MIB/MS kinome profiling data showed that, as expected, both Bcr-Abl and Lyn were potently inhibited by ponatinib treatment (S2 Fig). Immunoblot analyses were performed on the same lysates to validate changes in Bcr-Abl and Lyn observed from the MIB/MS data (Fig 2C). Short-term (1 hour) ponatinib treatment of MYL-R cells suppressed both Bcr-Abl and Lyn activity as determined by immunoblots of activating phosphorylation sites on Bcr-Abl, the Bcr-Abl substrates Crkl and Cbl, Src Family kinases, and Lyn substrates Crkl and Cbl (Fig 2C). Total BIRC6 and Mcl-1 proteins were not affected by short-term treatment (Fig 2D). Immunoblotting detected both the anti-apoptotic (Mcl-1_L_) and the pro-apoptotic (Mcl-1_S_) isoforms that result from differential splicing [60]. Only the anti-apoptotic isoform, Mcl-1_L_, was included for simplicity. Titanium enrichment and phosphopeptide analysis revealed ~4000 unique phosphopeptides. Phosphopeptide abundance was calculated using label-free quantification with MaxQuant [61], and normalized to MYL cells. A number of phosphopeptides determined to be enriched in MYL-R lysates decreased after ponatinib treatment (Herring, unpublished data), consistent with the change in Lyn activity in these cells (Fig 2C). From the list of phosphorylated proteins that followed the Lyn activity pattern, we identified a phosphopeptide [LEGDSDDLLEDS(480)DS(482)EEHS(486)R] from the anti-apoptotic protein BIRC6 that was multiply phosphorylated on serines 480, 482, and 486, proximal to the BIR domain (Fig 2A). Quantitation of the data showed that there was ~ a 1.6-fold increase in the BIRC6 phosphopeptide in MYL-R lysate compared to MYL lysate, and that ponatinib treatment of MYL-R cells reduced phosphopeptide abundance to levels close to those observed in MYL cells (Fig 2B).

We next examined BIRC6 mRNA expression in MYL and MYL-R cells, and compared these to another CML cell line, K562. Approximately equal numbers of MYL, MYL-R, and K562 cells were collected, and each cell population split into two parts, one for total RNA extraction and the other for immunoblot analysis. Total RNA was extracted and purified using RNeasy Mini Kit (Qiagen), and analyzed by QRT-PCR using BIRC6 and Mcl-1 TaqMan probes as described in Materials and Methods. Similarly, lysates were probed for BIRC6 protein by immunoblotting. Our data showed that BIRC6 and another anti-apoptotic protein known to be upregulated in MYL-R cells [29], Mcl-1, mRNA were significantly higher (*p* < 0.05) both in the imatinib-resistant MYL-R and K562 cell lines as compared to the imatinib-sensitive MYL cell line (Fig 2F). Similarly, Mcl-1 and BIRC6 protein levels were higher in MYL-R and K562 cells respectively (Fig 2E).

### Knockdown of BIRC6 in MYL-R cells increases sensitivity to imatinib

**Fig 3:**
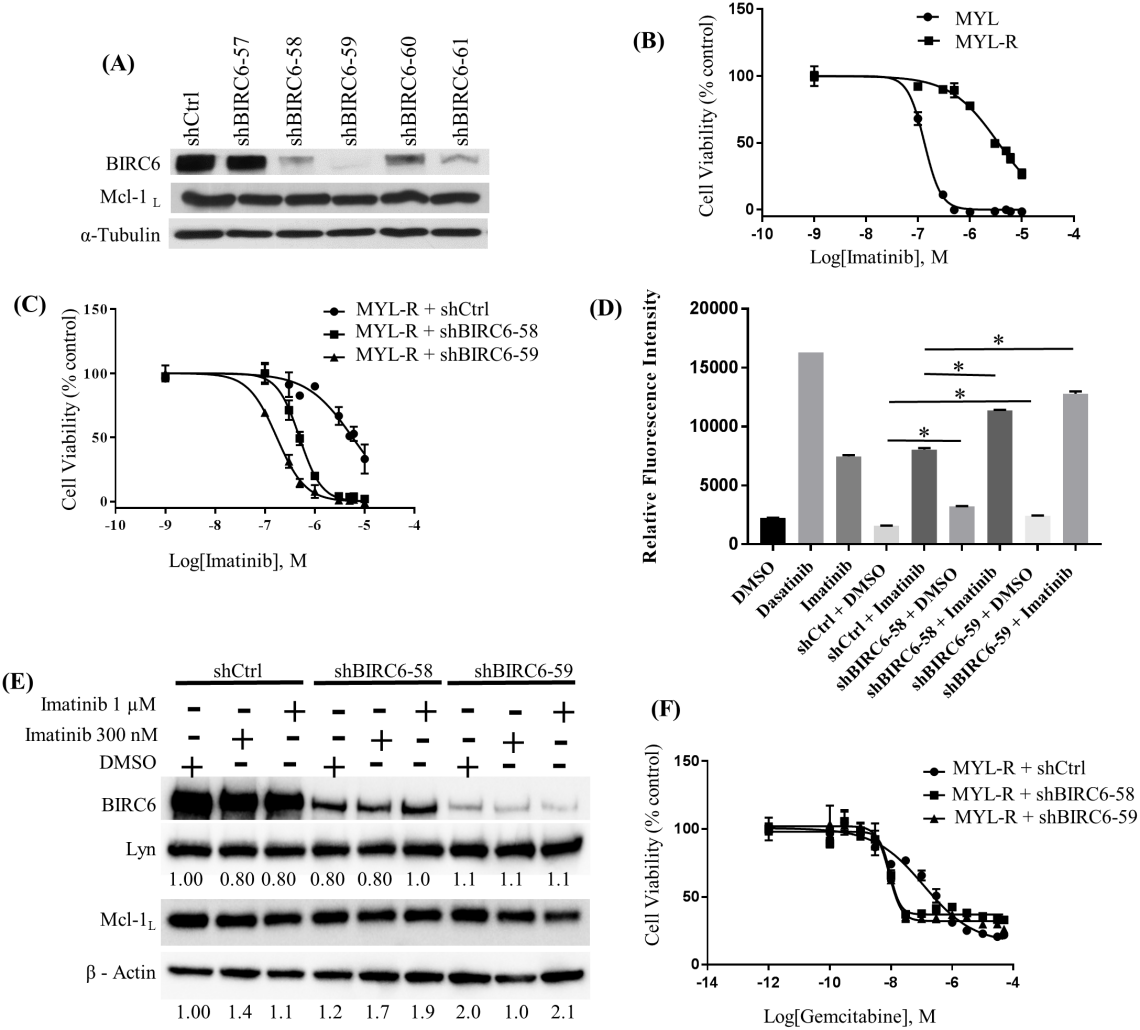
BIRC6 mediates drug resistance in MYL-R cells independently of Mcl-1. (A) Anti-BIRC6 shRNAs were used to knock down BIRC6 in MYL-R cells. Immunoblot analyses showed that shBIRC6-58, -59, and -61 yielded efficient knockdown without affecting Mcl-1. (B) MYL-R cells were resistant to imatinib (IC50 ~3.0 μM) as compared to MYL cells (IC50 ~0.2 μM), and (C) BIRC6 knockdown sensitized MYL-R cells to imatinib (IC50 ~0.2 μM). MYL, MYL-R, and BIRC6 knockdown MYL-R cells were cultured in triplicate in 96-well plates with increasing concentrations of imatinib for 72 hours, and cell viability was assessed by MTS assay. (D) Treatment of BIRC6 knockdown MYL-R cells with imatinib showed elevation in caspase-3/7 activity. BIRC6 knockdown MYL-R cells were treated with 1 μM imatinib for 24 hours. Parental MYL-R cells were treated with DMSO or 1 nM dasatinib or 1 μM imatinib. Caspase-3/7 activity was measured using a fluorogenic substrate as described in Materials and Methods. (E) BIRC6 knockdown or imatinib treatment in MYL-R cells did not affect total Lyn or total Mcl-1 protein levels. BIRC6 knockdown MYL-R cells were treated with DMSO or 300nM or 1 μM imatinib (IM) for 24 hours, and immunoblot analyses used to measure total Lyn and total Mcl-1 proteins. (F) Knockdown of BIRC6 in MYL-R cells increased sensitivity to gemcitabine. BIRC6 knockdown MYL-R cells were cultured as described in (C) with increasing concentrations of gemcitabine for 72 hours and cell viability determined by MTS assay. The data presented in this figure are representative of at least three independent experiments, and * represents *p < 0*.*05*.

Increased levels of BIRC6 have been observed in a number of human cancers and shown to correlate with poor prognostic profiles and higher incidences of disease relapse [19,21,23,55]. Consistent with this, we found higher levels of BIRC6 in MYL-R cells (Fig 2E and 2F). Thus, we examined whether imatinib had any effect on BIRC6 protein. MYL-R cells were treated for 24 hours with increasing concentrations of imatinib. A separate population of MYL-R cells was treated with 10 nM ponatinib. Immunoblotting showed that imatinib treatment was effective in reducing BIRC6 protein only in the micro-molar range with 50% reduction at 10 μM. Phospho-Bcr-Abl and the Bcr-Abl substrate, phospho-Crkl, were similar reduced. Ponatinib, on the other hand, reduced BIRC6 protein by 60% and almost completely reduced phospho-Bcr-Abl and phospho-Crkl (S3A Fig). Based on the above data, we investigated whether imatinib-resistance was dependent on BIRC6 expression in MYL-R cells. We infected MYL-R cells with lentiviral particles containing five independent anti-BIRC6 shRNAs or shCtrl. Stably transduced cells were selected with puromycin, and BIRC6 protein levels were measured by immunoblotting (Fig 3A). These analyses showed that shRNA constructs shBIRC6-58 and shBIRC6-59 were the most efficient, reducing BIRC6 protein by approximately 90%. Thus all subsequent experiments used these constructs to achieve BIRC6 knockdown. Knockdown of BIRC6 did not affect Mcl-1 or total Lyn protein levels in MYL-R cells (Fig 3A and 3E). Further immunoblot analysis showed that while there was not a substantial decrease in either phospho-Crkl or total Crkl in BIRC6 knockdown MYL-R cells, phospho-Bcr-Abl and total Bcr-Abl were substantially reduced (S3B Fig). This was consistent with a previous study that showed enhanced degradation of Bcr-Abl upon disruption of survivin (BIRC5) in K562 cells [9].

To examine the effects of BIRC6 knockdown on imatinib resistance, we first examined the effects of imatinib on cell viability using an MTS assay.

As expected, MYL and MYL-R cells showed substantial differences in their imatinib sensitivities with IC50 values of ~ 0.2 μM and ~3.0 μM for MYL and MYL-R cells respectively (Fig 3B). Next, BIRC6-knockdown MYL-R cells were incubated with increasing concentrations of imatinib for 72 hours and MTS assay performed. BIRC6 knockdown resulted in ~15-fold shift (IC50 ~0.2 μM) in the imatinib dose-response curve and increased imatinib sensitivity to levels similar to that observed for MYL cells (IC50 ~0.2 μM) (Fig 3C). Importantly, increases in imatinib sensitivity resulting from BIRC6 knockdown were consistent with the level of BIRC6 knockdown achieved by the individual shRNAs with shBIRC6-59 resulting in greater sensitivity, further emphasizing BIRC6’s role in mediating imatinib resistance in MYL-R cells.

We next examined whether the effect of imatinib on viability of BIRC6 knockdown cells was specifically due to the induction of apoptosis. The stable BIRC6-knockdown MYL-R cell lines were treated with 1 μM imatinib for 24 hours and caspase-3/7 activity assays performed as described in Materials and Methods (Fig 3D). Data from the 24-hour treatment were reported because caspase-3/7 activity was determined to be maximal at this time-point. MYL-R parental cells (positive control) were treated with dasatinib, a dual Bcr-Abl and Src family kinase inhibitor known to induce caspase-3/7 activity in this cell line [14]. BIRC6 knockdown (shBIRC6-58 and -59) caused ~ 2-fold increase in caspase-3/7 activity compared to shCtrl. Compared to shCtrl, knockdown of BIRC6 (shBIRC6-58 and -59) significantly (*p* < 0.05) increased MYL-R sensitivity to imatinib (Fig 3D). Treatment of parental MYL-R cells and BIRC6 knockdown control cells with imatinib showed comparable caspase-3/7 activation. These observations demonstrate that BIRC6 reduces apoptosis in MYL-R cells treated with imatinib.

To further characterize the response of MYL and MYL-R cells to imatinib, we treated cells with 1 μM imatinib and measured caspase-3/7 activities at 0, 6, 12, 24, 48 and 72-hr time points (S3C Fig). The MYL cells yielded an early (~12 hours) peak response to imatinib that gradually subsided. On the other hand, MYL-R cells showed a slow, gradual response to imatinib with a lower peak response at about 48 hours. Moreover, consistent with lower levels of BIRC6 in MYL cells, MYL cells had a higher basal caspase-3/7 activity than the MYL-R cells (S3C Fig).

MYL-R cells express increased amounts of the anti-apoptotic protein Mcl-1 relative to MYL cells [14,29]. Therefore, we investigated whether the increase in imatinib sensitivity observed in BIRC6 knockdown MYL-R cells (Fig 3C) was due to concomitant loss of Mcl-1 protein. Separate populations of the stable BIRC6-knockdown MYL-R cell lines were treated with DMSO or imatinib (300 nM or 1 μM) for 24 hrs. MYL-R cells expressing shCtrl were similarly treated and immunoblot analyses performed (Fig 3E). The data showed that treatment of BIRC6-knockdown MYL-R cells with imatinib (300 nM or 1 μM) had no effect on Mcl-1 protein, further suggesting that the anti-apoptotic effects of BIRC6 in these cells are independent of Mcl-1.

We have also observed that MYL-R cells are resistant to cytotoxic nucleoside analogs like gemcitabine and ARA-C (Data not shown). Therefore, we examined whether BIRC6 knockdown affected sensitivity of MYL-R cells to gemcitabine using an MTS assay (Fig 3F). BIRC6-knockdown resulted in ~13-fold shift in the gemcitabine dose-response curve and significantly increased (*p* < 0.05) gemcitabine sensitivity (shCtrl, IC50 ~ 120 nM; shBIRC6-58, IC50 ~ 9 nM; shBIRC6-59, IC50 ~ 9 nM). Gemcitabine did not achieve absolute cell death under these experimental conditions likely due to its cytostatic mechanism of action [62]. Thus, BIRC6 mediates resistance to multiple anti-cancer drugs including targeted inhibitors and cytotoxic compounds.

### Lyn kinase regulates BIRC6 expression

**Fig 4:**
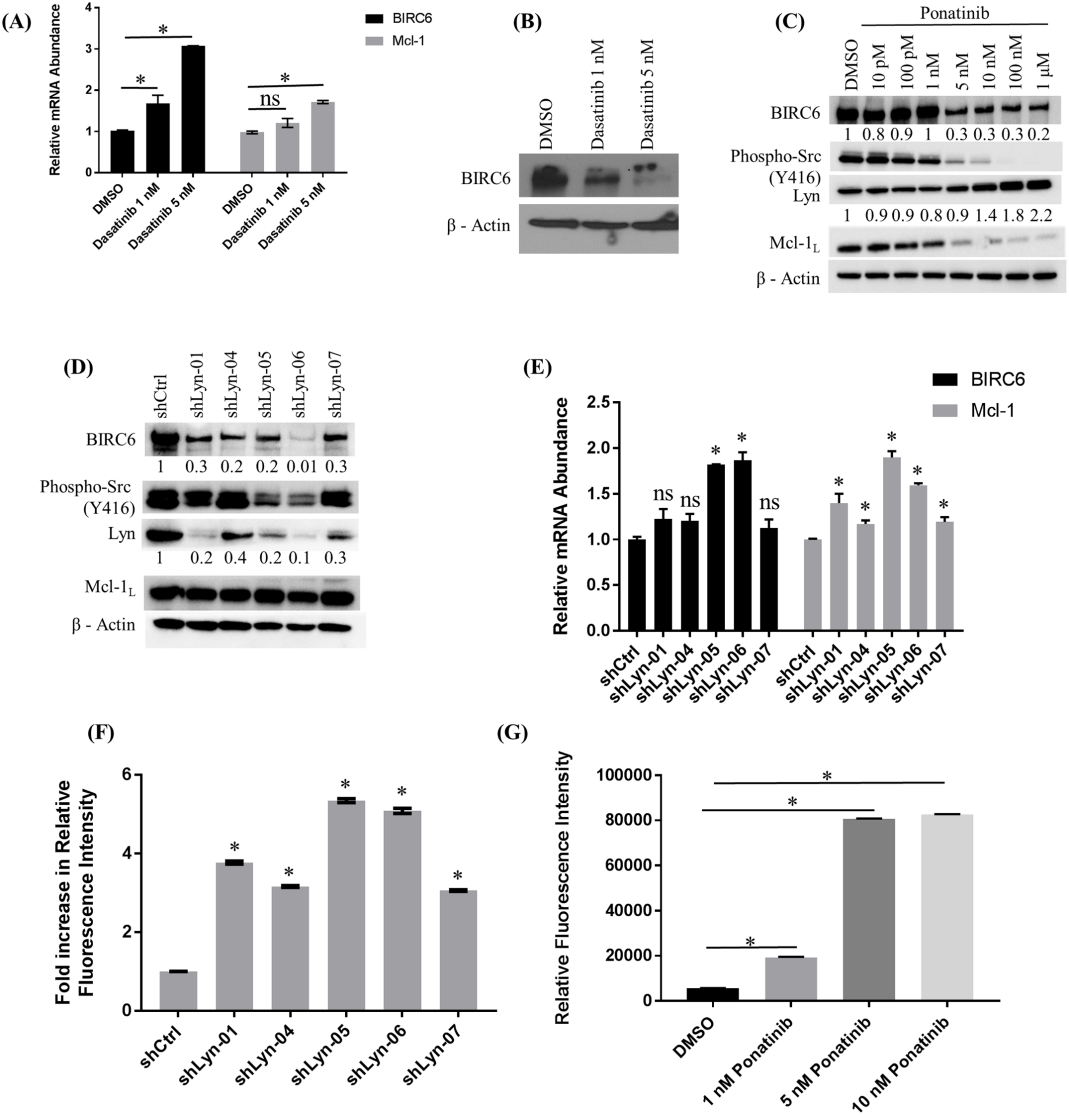
Lyn kinase regulates BIRC6 expression. (A) Treatment of MYL-R cells with dasatinib significantly increased BIRC6 and Mcl-1 mRNA levels as determined by QRT-PCR. MYL-R cells were treated with dasatanib (1 nM or 5 nM, 24 hours) and BIRC6 and Mcl-1 mRNA levels measured by QRT-PCR. (B) Immunoblotting of lysates from the same cells showed that dasatinib treatment reduced BIRC6 protein in MYL-R cells in a dose-dependent manner. (C) Ponatinib treatment of MYL-R cells recapitulated BIRC6 protein reduction observed with dasatinib. MYL-R cells were treated for 24 hours with increasing concentrations of ponatinib and immunoblot analyses used to measure BIRC6, Mcl-1, Lyn, and phospho-Src family (Y416) protein levels. Whereas BIRC6, phospho-Src family (Y416), and Mcl-1 were reduced in a dose-dependent manner, total Lyn was increased. (D) Whereas shRNA knockdown of Lyn in MYL-R cells substantially reduced BIRC6 protein, but not Mcl-1, (E) both BIRC6 and Mcl-1 mRNA levels were significantly increased by the more efficient Lyn knockdown shRNA constructs (shLyn-05 and -06). MYL-R cells were infected with lentiviral particles containing shRNA directed against Lyn. Upon selection of stably transduced cells, BIRC6 and Mcl-1 protein and mRNA levels were measured by immunoblotting and QRT-PCR. (F) Lyn knockdown in MYL-R cells was sufficient to increase caspase-3/7 activity. Data was normalized to shCtrl. (G) Ponatinib treatment of MYL-R cells significantly increased caspase-3/7 activity. MYL-R cells were treated with 1 nM, 5 nM, or 10 nM ponatinib or 0.1% DMSO for 24 hours and caspase-3/7 activity measured. The data are representative of three independent experiments, and * represents *p < 0*.*05*.

MYL-R cells are characterized by the overexpression and activation of Lyn kinase which is important for their survival [14,15,29]. Lyn activity is inhibited by dasatinib, a small molecule inhibitor of Bcr-Abl and other tyrosine kinases [35,36]. To examine if Lyn regulates BIRC6 expression, MYL-R cells were treated with dasatinib (1 or 5 nM) for 24 hours and BIRC6 mRNA and protein were analyzed using QRT-PCR and immunoblotting respectively (Fig 4A and 4B). Whereas dasatinib significantly increased (*p* < 0.05) BIRC6 mRNA in MYL-R cells, BIRC6 protein was substantially reduced (Fig 4A and 4B). Similarly, treatment of MYL-R cells with ponatinib, a more selective inhibitor of Bcr-Abl and Lyn [63,64] (S2 Fig), recapitulated the effects on BIRC6 protein (Fig 4C). Ponatinib substantially inhibited Lyn activity as measured by autophosphorylation of Src family kinases including Lyn (PY416) (Fig 4C), and in parallel, reduced both BIRC6 and Mcl-1. To further validate the importance of Lyn in regulating BIRC6, we examined the effects of Lyn-knockdown in MYL-R cells. We tested five lentiviral Lyn targeted shRNA oligonucleotides and a non-targeting shRNA (shCtrl). Aliquots were harvested from each of the cell lines, and QRT-PCR and immunoblot analyses performed (Fig 4E and 4D). Anti-Lyn shRNA constructs shLyn-01, shLyn-05, and shLyn-06 were the most efficient in knocking down Lyn as determined by immunoblotting, reducing Lyn protein by 80–90%. A substantial reduction in BIRC6 protein was observed in response to Lyn knockdown (Fig 4D). By contrast, shRNA knockdown of Lyn did not affect Mcl-1 protein (Fig 4D). Loss of BIRC6 protein was not mediated by changes in gene expression as mRNA levels were significantly (p < 0.05 for shLyn-05 and -06) elevated in response to Lyn knockdown (Fig 4E). Similarly, Mcl-1 mRNA levels were significantly increased in response to Lyn knockdown. These data suggest that Lyn regulates BIRC6 protein at the translational or post-translational level.

Since Lyn inhibition or knockdown reduced BIRC6 protein levels in MYL-R cells (Fig 4C and 4D), we investigated whether this was sufficient to increase caspase-3/7 activity in these cells. Lyn knockdown cells were harvested and caspase-3/7 activity was measured (Fig 4F). Lyn knockdown resulted in significant increase (*p* < 0.05) in caspase-3/7 activity in MYL-R cells. Cells expressing the more efficient Lyn-knockdown oligonucleotides (shLyn-05 and shLyn-06), showed a ~5-fold increase in caspase-3/7 activity. We next examined if a change in the mitochondrial membrane potential contributed to the increase in caspase-3/7 activity in the Lyn-knockdown cells. Approximately 1x10^6^ of the Lyn-knockdown or control MYL-R cells were analyzed for mitochondrial membrane potential and caspase-3/7 activity using MitoCasp^™^ (S4A Fig). Lyn knockdown lowered the mitochondrial membrane potential and increased caspase-3/7 activity. Consistent with the Lyn knockdown immunoblot data, the best anti-Lyn oligonucleotides yielded the largest reduction in mitochondrial membrane potential and increase in caspase-3/7 activity. Furthermore, the most efficient anti-Lyn shRNA construct (shLyn-06) yielded the highest percent of apoptotic cells as determined by the fraction of cells in quadrant 3 (Q3) (S4B Fig). Based on the above observations, we investigated whether inhibiting Lyn with ponatinib would affect the basal level of caspase-3/7 activity. MYL-R cells were treated with ponatinib (1 nM or 5 nM or 10 nM) or 0.1% DMSO for 24 hours and caspase-3/7 activity assays performed (Fig 4G). Compared to DMSO, there was significant increase (*p* < 0.05) in basal caspase-3/7 activity with ~ 2-fold increase upon treatment with 1 nM ponatinib and ~ 8-fold increase upon treatment with 5 nM or 10 nM ponatinib. These observations support the importance of Lyn-mediated regulation of BIRC6 in MYL-R cells.

**Fig 5:**
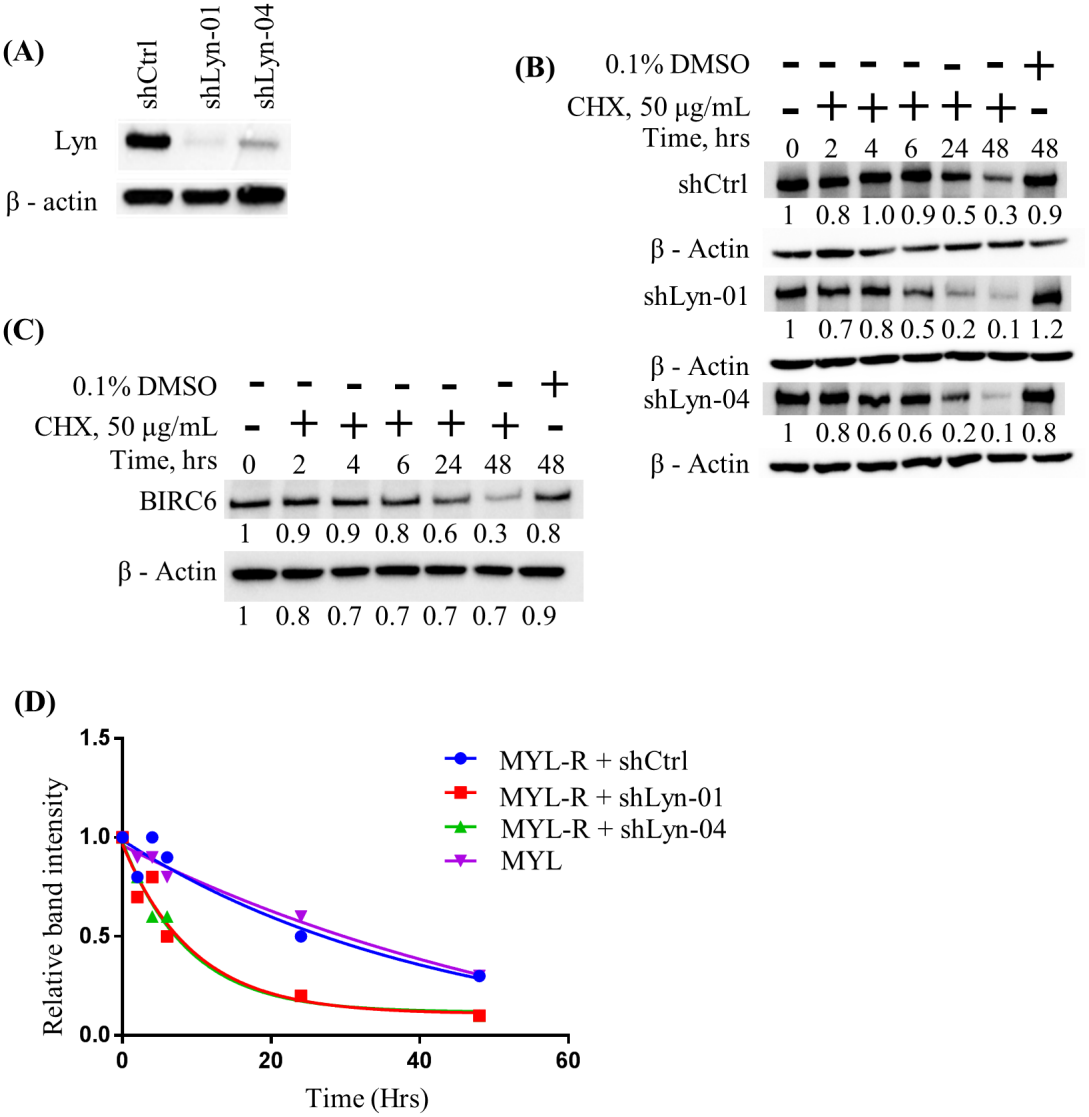
Lyn knockdown reduced the half-life of BIRC6 protein in MYL-R cells. (A) MYL-R cells were infected with lentiviral particles containing anti-Lyn shRNA constructs (shLyn-01 and -04), and Lyn knockdown confirmed by immunoblot analysis. (B) Lyn knockdown in MYL-R cells reduced the half-life of BIRC6 4-fold (~24 hrs in shCtrl cells to ~ 6 hrs in Lyn knockdown cells). Lyn knockdown MYL-R cells were incubated with 50 μg/mL cycloheximide (CHX) in a time-course manner and BIRC6 protein determined by immunoblotting. (C) BIRC6 half-life in MYL cells was ~24 hours. MYL cells were treated with CHX as in (B) and BIRC6 protein determined by immunoblotting. (D) GraphPad^™^ Prism was used to plot change in BIRC6 stability over time upon Lyn-knockdown in MYL-R cells as described in (B). The BIRC6 stability data presented here are representative of two independent experiments.

We next investigated if the observed reduction in BIRC6 protein levels in MYL-R cells upon Lyn inhibition or Lyn knockdown was through effects on BIRC6 protein stability. Two MYL-R cell populations infected with anti-Lyn shRNAs (shLyn-01 and shLyn-04) (Fig 5A) were selected and treated with cycloheximide (50 μg/mL). Cells were harvested at 0, 2, 4, 6, 24, and 48 hours after cycloheximide treatment, and immunoblotted for BIRC6 (Fig 5B). We observed that BIRC6 protein half-life was approximately the same (~24 hours) in shCtrl-infected MYL-R cells as that in the parental MYL cells (Fig 5B and 5C). By contrast, Lyn knockdown caused a substantial reduction in BIRC6 protein stability, with the protein half-life reduced ~4-fold to approximately 6 hours (Fig 5B and 5D).

### CDK9 regulates BIRC6 mRNA levels

**Fig 6:**
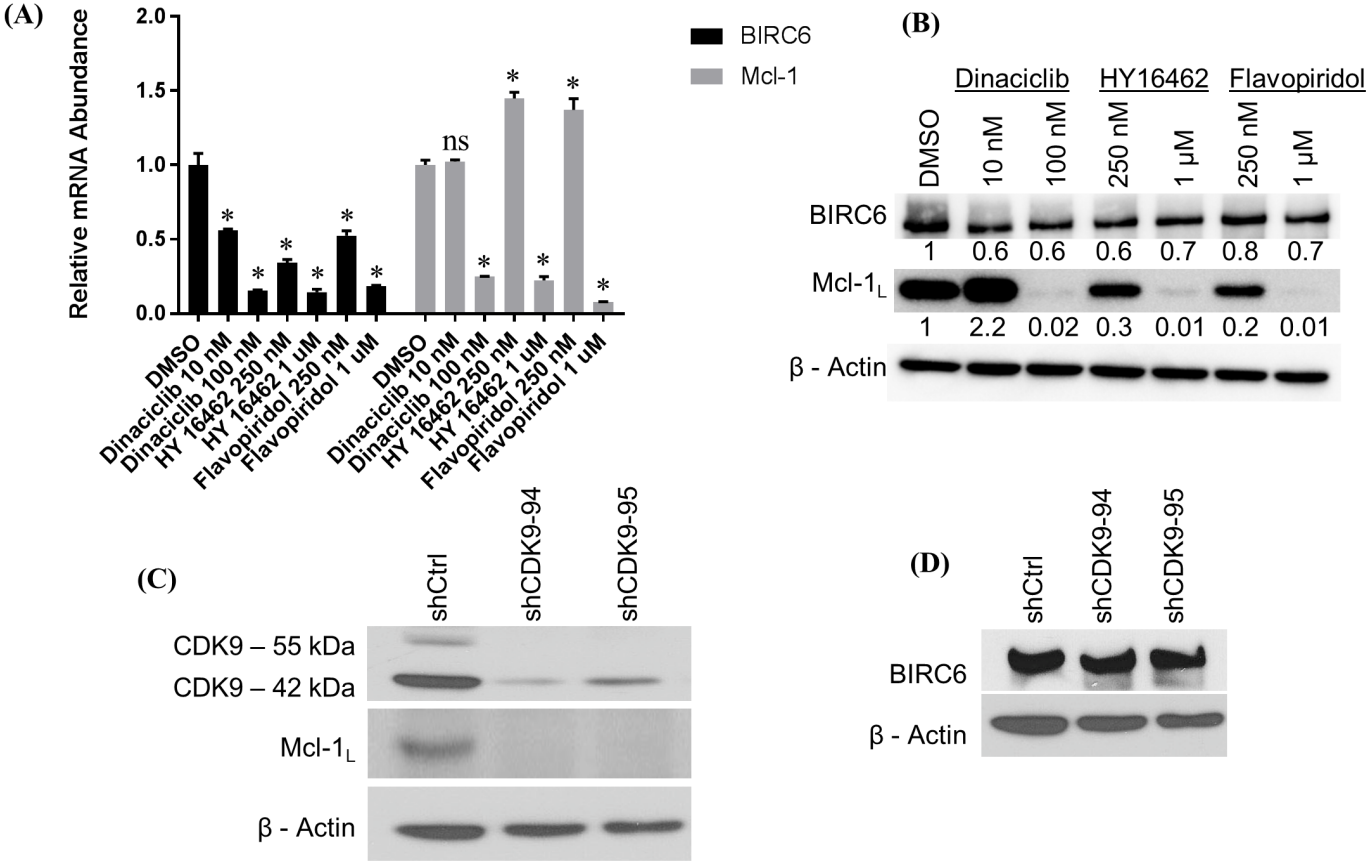
CDK9 regulates BIRC6 mRNA levels. (A) Inhibition of CDK9 with dinaciclib, HY-16462, and flavopiridol significantly reduced BIRC6 and Mcl-1 mRNA levels in a dose-dependent manner. MYL-R cells were treated with dinaciclib (10 or 100 nM), HY-16462 (250 nM or 1 μM), and flavopiridol (250 nM or 1 μM) for 24 hours and BIRC6 and Mcl-1 mRNA measured by QRT-PCR. (B) Immunoblot analyses of lysates from the same conditions in (A) above had no substantial effect on BIRC6 protein. By contrast, Mcl-1 protein levels were reduced in a dose-dependent manner except with 10 nM dinaciclib that showed a substantial increase in Mcl-1 protein. (C and D) shRNA knockdown of CDK9 in MYL-R cells and immunoblot analysis recapitulated the data obtained in (B) above. The data presented here are representative of three independent experiments. * Represents *p < 0*.*05*.

We previously showed that increased Lyn activity correlated with elevated Mcl-1 expression in MYL-R cells [29]. Other studies demonstrated that Cyclin-dependent kinase 9 (CDK9), a critical component of the elongation factor complex P-TEFb, is required for transcription of Mcl-1 [30,37]. CDK9 regulates RNA Polymerase II (RNA Pol II) by phosphorylating it, thereby promoting transcription elongation [65]. Cancer cells require continuous activity of RNA Pol II to suppress oncogene-induced apoptosis [66,67]. Small molecule inhibitors of CDK9 have been shown to induce apoptosis in cancer cells by suppressing Mcl-1 [30,37]. To investigate if CDK9 regulates BIRC6, we treated MYL-R cells with the CDK9 inhibitors dinaciclib, flavopiridol, and a novel CDK9 inhibitor (HY-16462), and compared Mcl-1 and BIRC6 expression. Whereas dinaciclib and flavopiridol target multiple CDKs [66,68], HY-16462 is reported to be a highly specific CDK9 inhibitor (Novartis, data not shown). All three CDK9 inhibitors significantly (*p < 0*.*05*) reduced BIRC6 mRNA in a dose-dependent manner with the highest concentrations yielding >5-fold reduction as determined by QRT-PCR (Fig 6A). Mcl-1 mRNA was similarly reduced. Immunoblot analysis revealed that the highest concentrations of CDK9 inhibitors strongly reduced Mcl-1 protein as previously reported [30,37,69] (Fig 6B). Consistent with loss in mRNA, we observed a decrease in BIRC6 protein after treatment with CDK9 inhibitors. The decrease in BIRC6 protein was modest compared to Mcl-1 but likely reflects differences in protein half-life. To further confirm the role of CDK9 in regulating BIRC6 expression, we used shRNA-mediated knockdown of CDK9 in MYL-R cells. Knockdown of CDK9 substantially reduced Mcl-1 protein levels (Fig 6C), but had no effect on BIRC6 (Fig 6D). These data demonstrate differential regulation of BIRC6 and Mcl-1 by CDK9 and suggest that specific inhibition of CDK9 is not sufficient to decrease BIRC6 protein levels.

### Lyn regulates caspase-mediated degradation of BIRC6 in MYL-R cells

**Fig 7:**
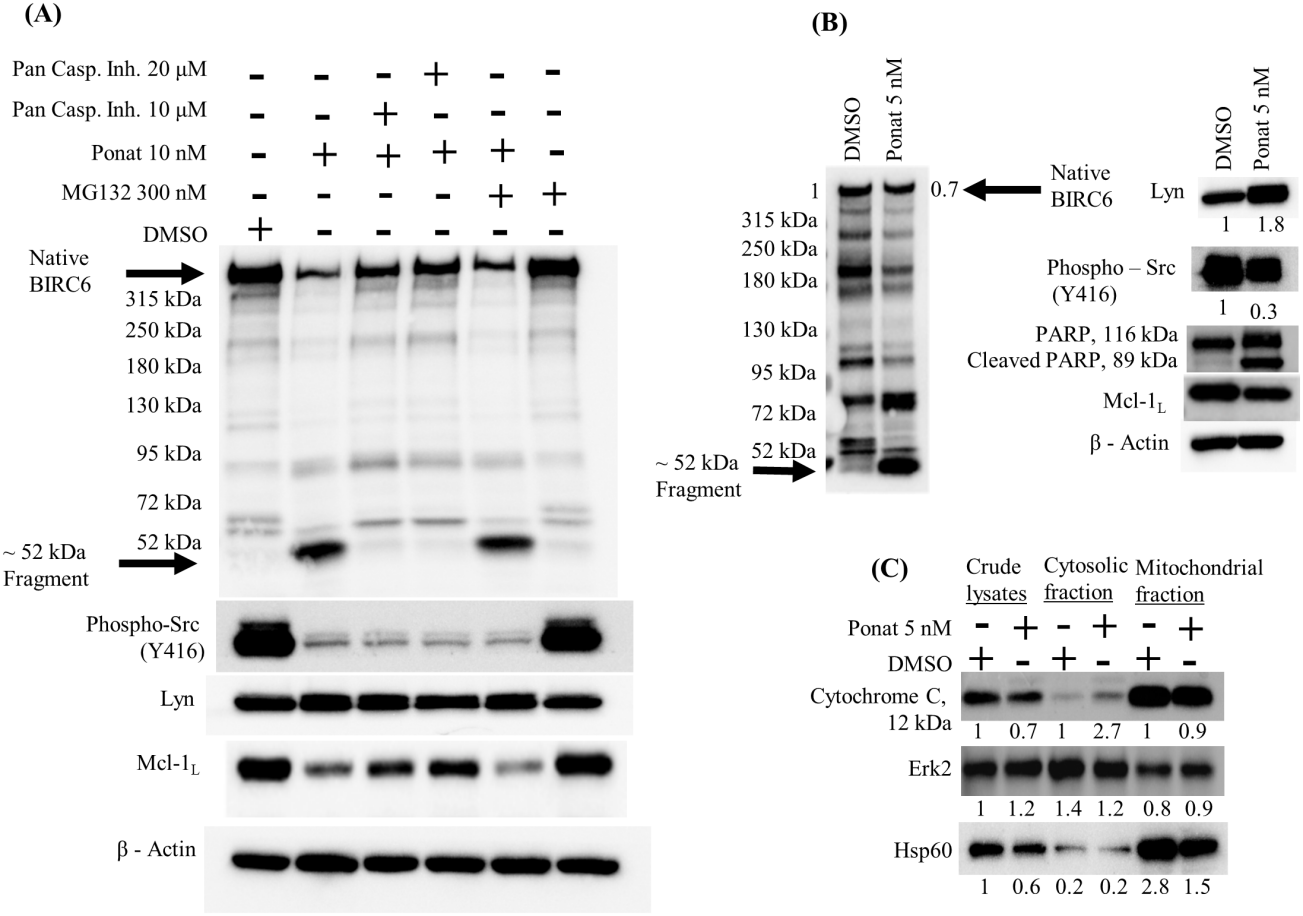
Lyn regulates caspase-mediated degradation of BIRC6 in MYL-R cells. (A) Caspase inhibitors, but not proteasome inhibitors inhibited ponatinib-mediated BIRC6 degradation. MYL-R cells were incubated with 10 nM ponatinib or 0.1% DMSO and/or a pan-caspase inhibitor (Z-VAD-FMK 10 or 20 μM) and/or the proteasome inhibitor MG132 (300 nM) for 24 hours and BIRC6 protein examined by immunoblotting. Inhibition of Lyn (or Src family kinases) was validated by the loss in phospho-Src family (Y416). (B) Inhibition of Lyn in MYL-R cells caused a 30% reduction in BIRC6 protein and induced PARP cleavage as determined by immunoblot analyses. MYL-R cells were incubated with 5 nM ponatinib or 0.1% DMSO for 24 hours and BIRC6 protein measured. Induction of apoptosis was demonstrated by PARP cleavage. (C) Inhibition of Lyn in MYL-R cells caused cytochrome c release from mitochondria. MYL-R cells were treated with 5 nM ponatinib or 0.1% DMSO for 24 hours and mitochondria enriched using the Cytochrome C Releasing Apoptosis Assay Kit. Cytochrome c release was measured by immunoblotting cytosolic and mitochondrial fractions. Erk2 and Hsp60 were used as cytosolic and mitochondrial markers respectively. The data presented here are representative of three independent experiments.

BIRC6 is degraded by caspases in response to cell death signals [20]. We observed multiple fragments of BIRC6 after treatment of MYL-R cells with ponatinib (10 nM, 24hr) or dasatinib (1 nM, 24hr), including formation of a prominent ~52-kDa band (Fig 7A). This fragment was determined to contain the N-terminal region of BIRC6 as confirmed by the location of the antibody epitope and peptide competition experiment (Fig 7A and data not shown). Interestingly, the formation of the BIRC6 ~52-kDa immunoreactive band was significantly reduced in MYL-R cells co-treated with a pan-caspase inhibitor, Z-VAD-FMK (Fig 7A), indicating that the pan-caspase inhibitor rescued BIRC6 loss, further emphasizing the role played by caspases in BIRC6 degradation. Others previously reported that the onset of apoptosis can lead to BIRC6 degradation by proteasome and caspase cleavage [46]. However, addition of a proteasome inhibitor (MG-132) had no effect on BIRC6 degradation in ponatinib-treated cells (Fig 7A). The data suggest that caspases are primarily involved in BIRC6 degradation following Lyn inhibition.

Previous studies showed that increased cell proliferation was accompanied with elevated tyrosine phosphorylation inside mitochondria mediated by activated Src family kinases [70]. Additionally, active Lyn was reported to accumulate in the mitochondrial inter-membrane space where it complexes with a multi-protein complex [70]. Hence, we investigated whether Lyn inhibition would trigger mitochondrial events leading to caspase activation and loss in BIRC6 protein. MYL-R cells were treated with 5 nM ponatinib or 0.1% DMSO and cytochrome c protein release analyzed by immunoblotting (Fig 7C). Ponatinib treatment resulted in 70% loss in Src family kinases activation (PY416) and 30% loss in BIRC6 protein (Fig 7B). Total Lyn was increased by 80%. The appearance of cleaved PARP in whole cell lysates from ponatinib-treated but not DMSO-treated cells confirmed the induction of apoptosis (Fig 7B). Immunoblot analysis of cytochrome c was performed on crude lysate, cytosolic, and mitochondrial fractions. Cytochrome c bands were normalized to the respective DMSO fractions. Lysates from MYL-R cells treated with ponatinib showed a 2.7-fold increase in cytosolic cytochrome c (Fig 7C). These data suggest that Lyn reduces BIRC6 degradation by regulating mitochondrial events that are key to caspase activation.

## Discussion

Acquired resistance to apoptosis is an important defining characteristic of many drug resistant cancers [15,57,71,72]. While there is considerable evidence supporting BIRC6’s role in mediating drug resistance in several human cancers, the mechanisms regulating its expression and stability are not well understood [21,23,45,52–55,71,72]. We used a model of imatinib-resistance in CML cells to investigate the molecular determinants of acquired resistance. Phosphoproteomics of MYL and MYL-R cells identified BIRC6 as a potential regulator of imatinib resistance in MYL-R cells. Our data demonstrate three important observations: BIRC6 mediates imatinib resistance in MYL-R cells; Lyn activity regulates BIRC6 expression and stability; and inhibition or knockdown of Lyn results in caspase-mediated degradation of BIRC6.

In our MYL-R model of imatinib-resistant CML, we observed Lyn activity to be correlated with increased phosphorylation, expression, and stability of BIRC6. Lyn inhibition with ponatinib resulted in loss of Mcl-1 protein, release of cytochrome c from mitochondria, caspase activation, and degradation of BIRC6 protein. Lyn depletion with shRNA also led to loss in BIRC6 protein. Regulation of BIRC6 degradation has been described to occur via various mechanisms including caspase-3, -6, -7, -8, and -9 cleavage [20]. We observed that induction of apoptosis correlated with both the loss of full length BIRC6 and the formation of a prominent N-terminal BIRC6 fragment by immunoblot analysis. Use of the pan-caspase inhibitor, Z-VAD-FMK, prevented both loss of full length BIRC6 and fragment formation indicating that caspase activation was required for BIRC6 degradation in response to Lyn inhibition. We did not observe any role for proteasome-mediated degradation of BIRC6 in response to Lyn inhibition as suggested by the failure of the proteasome inhibitor, MG-132, to rescue BIRC6 degradation.

Our lab previously made a link between Lyn activity and increased Mcl-1 [29]. In the present study, we show that BIRC6-knockdown rendered MYL-R cells several-fold more sensitive to imatinib and gemcitabine, two compounds used in cancer therapy. The increase in imatinib sensitivity after BIRC6 knockdown occurred without concomitant loss of Mcl-1. This was surprising since multiple studies have suggested that Mcl-1 is necessary for mediating drug resistance in cancers including leukemias [29,30,33,34,37,69]. Mcl-1 and CDK9 inhibitors, however, have had limited success in cancer therapy [40,41,66,73–75]. Our data show that BIRC6 exerts a dominant role in mediating imatinib resistance independently of Mcl-1.

Our study suggests that BIRC6 expression and stability in MYL-R cells is dependent on Lyn and that elevated BIRC6 levels promotes cell survival. The phosphorylated peptide derived from BIRC6 correlated with Lyn activity, exhibiting increased levels in MYL-R relative to MYL cells and decreased levels after ponatinib treatment. Notably, short-term ponatinib treatment did not affect total BIRC6 protein suggesting that the change in BIRC6 phosphopeptide abundance was largely dependent on changes in kinase signaling. Interestingly, this peptide contains consensus caspase-8/9 and -3/7 cleavage sites that overlap with potential consensus phosphorylation motifs of certain serine/threonine kinases including CK2, PLK1, Aurora A kinase, and others [76–81]. Treatment of MYL-R cells with a CK2 inhibitor caused a substantial reduction in BIRC6 protein (S5A Fig). Furthermore, BIRC6 immunoprecipitation experiments showed that CK2 co-precipitated with BIRC6 (S5B and S5C Fig) as reported earlier [82]. CK2 phosphorylation of caspase substrates in the vicinity of caspase recognition motifs protects proteins from caspase cleavage [76]. Interestingly, the ~52-kDa N-terminal degradation fragment observed in our studies (Fig 7A) is consistent with the predicted molecular weight of the product of caspase cleavage near the identified phosphorylation sites proximal to the BIR domain. Cleavage of BIRC6 in the N-terminal region inactivates its ability to bind and inhibit caspases [20]. Thus, phosphorylation of BIRC6 by CK2 may prevent BIRC6 degradation by caspases. While previous studies suggested that Lyn regulates CK2 activity in imatinib-resistant CML cells [83], we did not observe differences in CK2 activity in MYL and MYL-R cells as measured by the phosphorylation of validated CK2 substrates [84], CK2β and EEF1D, (S5D Fig). It remains to be determined if CK2 regulates BIRC6 stability through phosphorylation of these sites.

In summary, our data demonstrate that BIRC6 is enriched in imatinib-resistant CML cells (MYL-R), and that knockdown of BIRC6 was sufficient to increase imatinib sensitivity independent of Mcl-1. We showed that CDK9 inhibition suppressed both BIRC6 and Mcl-1 mRNA and protein levels. The increase in Mcl-1 protein caused by dinaciclib (10 nM, 24 hours) was likely due to activation of compensatory signaling mechanisms but was not pursued further in the present study. We next used anti-CDK9 shRNAs to test whether CDK9 specifically regulates BIRC6. Whereas Mcl-1 was reduced by >95% upon CDK9 knockdown, there was no effect on BIRC6 protein suggesting that CDK9 does not regulate BIRC6 protein. These data indicate that the effects observed with CDK9 inhibitors were likely due to off-target effects rather than specific inhibition of CDK9. CDK9 inhibitors, including those used in this study, have been reported both by their manufacturers and in the literature to have poor specificity for CDK9 [66,68,85]. Thus, CDK9 inhibitors are unlikely to overcome imatinib resistance mediated by BIRC6. Taken together, these data also indicate that targeting Mcl-1 directly (Obatoclax Mesylate) or indirectly through CDK9 inhibition will have little effect on drug resistance in cells expressing high levels of BIRC6. Although there are currently no direct inhibitors of BIRC6, targeting Lyn or kinases that maintain BIRC6 expression or stability may have therapeutic potential in treating drug resistant CML or other cancers.

## Supporting information

S1 Fig Ponatinib is effective against imatinib-resistant CML cells (MYL-R). MYL and MYL-R cells were cultured in triplicate in 96-well plates with increasing concentrations of ponatinib for 72 hours, and cell viability was assessed by MTS assay. MYL and MYL-R showed no difference in ponatinib sensitivity with IC50 values of ~1.3 nM and ~1.2 nM respectively.
(PDF)

S2 Fig MIB/MS analysis of lysates from MYL-R cells treated with ponatinib revealed select kinase inhibition. MYL-R cells were treated with 10 nM ponatinib or DMSO for 1 hour and kinome changes analyzed by MIB/MS in two independent experiments. A total of ~ 230 kinases were identified in each of the two experiments. Kinases were quantified by label-free quantification using the MAXQUANT software package with integrated search engine (ANDROMEDA). Data is representative of results from the two experiments, and represent changes in the kinase abundance as determined from ratios of ponatinib/DMSO. Ratios are defined by the dashed lines where <1 and >1 respectively denote decreased and increased MIB binding of kinase in lysates from ponatinib-treated versus DMSO-treated MYL-R cells.
(PDF)

S3 Fig (A) Ponatinib more effectively suppressed Bcr-Abl and Lyn signaling, and BIRC6 protein than imatinib. MYL-R cells were treated with increasing concentrations of imatinib or 10 nM ponatinib or 0.1% DMSO for 24 hours and immunoblot analyses performed to examine the effects on BIRC6, phospho-Bcr-Abl, and Bcr-Abl/Lyn substrate, Crkl. (B) BIRC6 knockdown in MYL-R cells did not affect either phospho-Crkl or total Crkl. By contrast, BIRC6 knockdown caused substantial decrease in both phospho-Bcr-Abl and total Abl. (C) MYL-R cells had delayed activation of caspase-3/7 in response to imatinib treatment relative to MYL cells. MYL and MYL-R cells were treated with 1 μM imatinib in a time-course manner: 0, 6, 12, 24, 48 and 72 hours. Treatment was scheduled so that all cells were harvested at the 72-hr time-point. Caspase-3/7 activity was measured for each condition using a fluorogenic assay. MYL cells showed a two-fold higher basal caspase-3/7 activity relative to MYL-R cells.
(PDF)

S4 Fig Lyn knockdown in Myl-R cells lowered mitochondrial membrane potential and increased caspase-3/7 activity. (A) Lyn knockdown resulted in lower membrane potential and increased caspase-3/7 activity in MYL-R cells as determined by flow cytometry. Knockdown of Lyn was achieved by infecting MYL-R cells with lentiviral particles containing shRNA directed against Lyn. Fluorescence intensities for mitochondrial membrane potential and caspase-3/7 activity for Lyn knockdown MYL-R cells (shCtrl, shLyn-01, shLyn-04, shLyn-05, shLyn-06, and shLyn-07) were measured using the MitoCasp^™^ dual sensor. (B) The most efficient anti-Lyn shRNA construct (shLyn-06) yielded the highest percent of apoptotic cells as determined by the fraction of cells in quadrant 3 (Q3).
(PDF)

S5 Fig (A) CK2 inhibition substantially reduced BIRC6 protein. MYL-R cells were treated with CX-4945, a small molecule inhibitor of CK2, in a time-course manner and cells harvested after 24, 48, and 72 hours. Immunoblot analyses were performed to examine BIRC6 protein and activation level of a validated CK2 substrate (phospho-IF2β). (B) BIRC6 was immunoprecipitated from lysates of MYL-R cells. The supernatant and beads-only lanes showed no BIRC6 protein as determined by immunoblot analysis, and (C) CK2 co-immunoprecipitated with BIRC6. CK2α was present in the BIRC6 IP but not in the beads-only control. **(D)** Baseline CK2 activity is the same in both MYL and MYL-R cells. MYL and MYL-R cells were lysed and immunoblot analyses performed to determine the activity level of CK2 (phospho-CK2β) and the level of active CK2 substrate (phospho-EEF1D). The data showed that CK2 activity was the same in MYL and MYL-R cells.
(PDF)
