# Supplementary material for: BIRC6 mediates imatinib resistance independently of Mcl-1
Source: PLoS One. 2017 May 16;12(5):e0177871. doi: 10.1371/journal.pone.0177871 (PMC5433768; doi:10.1371/journal.pone.0177871)
Supplement: S1 Fig — MYL and MYL-R cells were cultured in triplicate in 96-well plates with increasing concentrations of ponatinib for 72 hours, and cell viability was assessed by MTS assay. MYL and MYL-R showed no difference in ponatinib sensitivity with IC50 values of ~1.3 nM and ~1.2 nM respectively. (PDF) [file pone.0177871.s001.pdf]

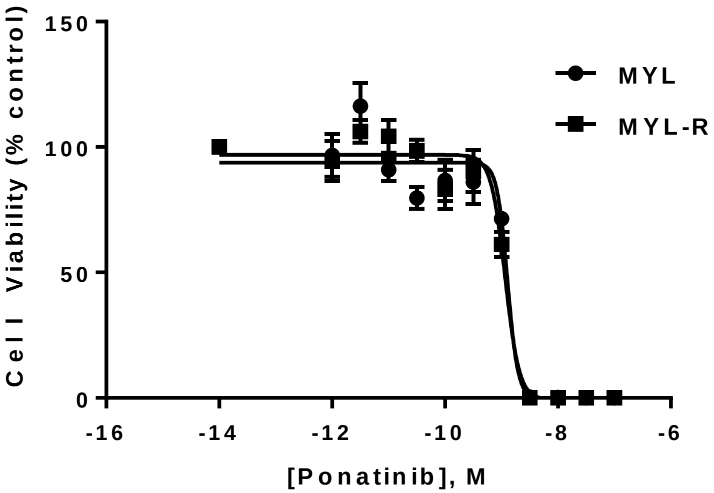

S1 Fig. Ponatinib is effective against imatinib-resistant CML cells (MYL-R).
MYL and MYL-R cells were cultured in triplicate in 96-well plates with increasing concentrations of ponatinib for 72 hours, and cell viability was assessed by MTS assay. MYL and MYL-R showed no difference in ponatinib sensitivity with IC50 values of ~1.3 nM and ~1.2 nM respectively.
