# Supplementary material for: BIRC6 mediates imatinib resistance independently of Mcl-1
Source: PLoS One. 2017 May 16;12(5):e0177871. doi: 10.1371/journal.pone.0177871 (PMC5433768; doi:10.1371/journal.pone.0177871)
Supplement: S2 Fig — MYL-R cells were treated with 10 nM ponatinib or DMSO for 1 hour and kinome changes analyzed by MIB/MS in two independent experiments. A total of ~ 230 kinases were identified in each of the two experiments. Kinases were quantified by label-free quantification using the MAXQUANT software package with integrated search engine (ANDROMEDA). Data is representative of results from the two experiments, and represent changes in the kinase abundance as determined from ratios of ponatinib/DMSO. Ratios are defined by the dashed lines where <1 and >1 respectively denote decreased and increased MIB binding of kinase in lysates from ponatinib-treated versus DMSO-treated MYL-R cells. (PDF) [file pone.0177871.s002.pdf]

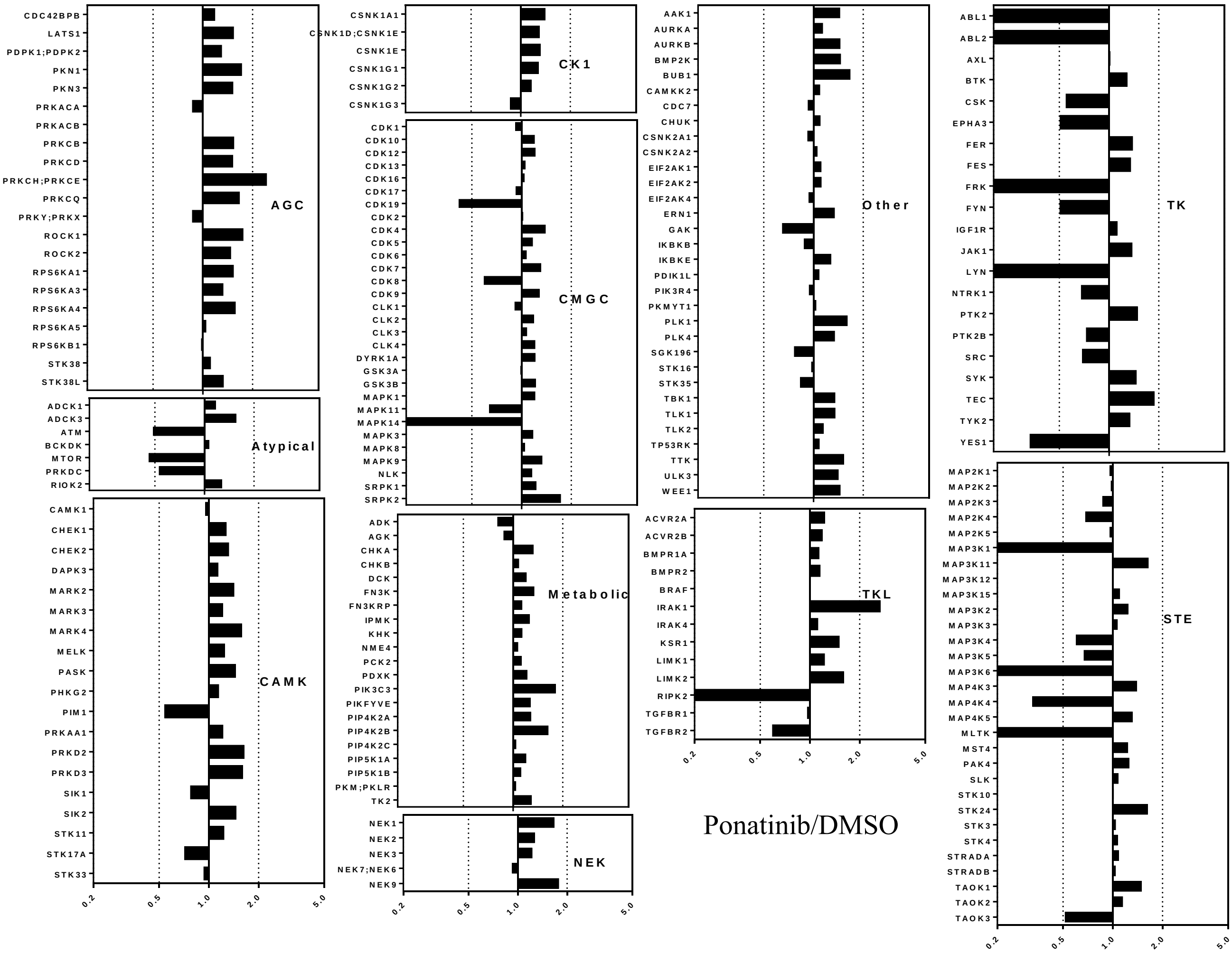

S2 Fig. MIB/MS analysis of lysates from MYL-R cells treated with ponatinib revealed select kinase inhibition. MYL-R cells were treated with 10 nM ponatinib or DMSO for 1 hour and kinome changes analyzed by MIB/MS in two independent experiments. A total of ~ 230 kinases were identified in each of the two experiments. Kinases were quantified by label-free quantification using the MAXQUANT software package with integrated search engine (ANDROMEDA). Data is representative of results from the two experiments, and represent changes in the kinase abundance as determined from ratios of ponatinib/DMSO. Ratios are defined by the dashed lines where <1 and >1 respectively denote decreased and increased MIB binding of kinase in lysates from ponatinib-treated versus DMSO-treated MYL-R cells.
