# Supplementary material for: BIRC6 mediates imatinib resistance independently of Mcl-1
Source: PLoS One. 2017 May 16;12(5):e0177871. doi: 10.1371/journal.pone.0177871 (PMC5433768; doi:10.1371/journal.pone.0177871)
Supplement: S4 Fig — (A) Lyn knockdown resulted in lower membrane potential and increased caspase-3/7 activity in MYL-R cells as determined by flow cytometry. Knockdown of Lyn was achieved by infecting MYL-R cells with lentiviral particles containing shRNA directed against Lyn. Fluorescence intensities for mitochondrial membrane potential and caspase-3/7 activity for Lyn knockdown MYL-R cells (shCtrl, shLyn-01, shLyn-04, shLyn-05, shLyn-06, and shLyn-07) were measured using the MitoCasp™ dual sensor. (B) The most efficient anti-Lyn shRNA construct (shLyn-06) yielded the highest percent of apoptotic cells as determined by the fraction of cells in quadrant 3 (Q3). (PDF) [file pone.0177871.s004.pdf]

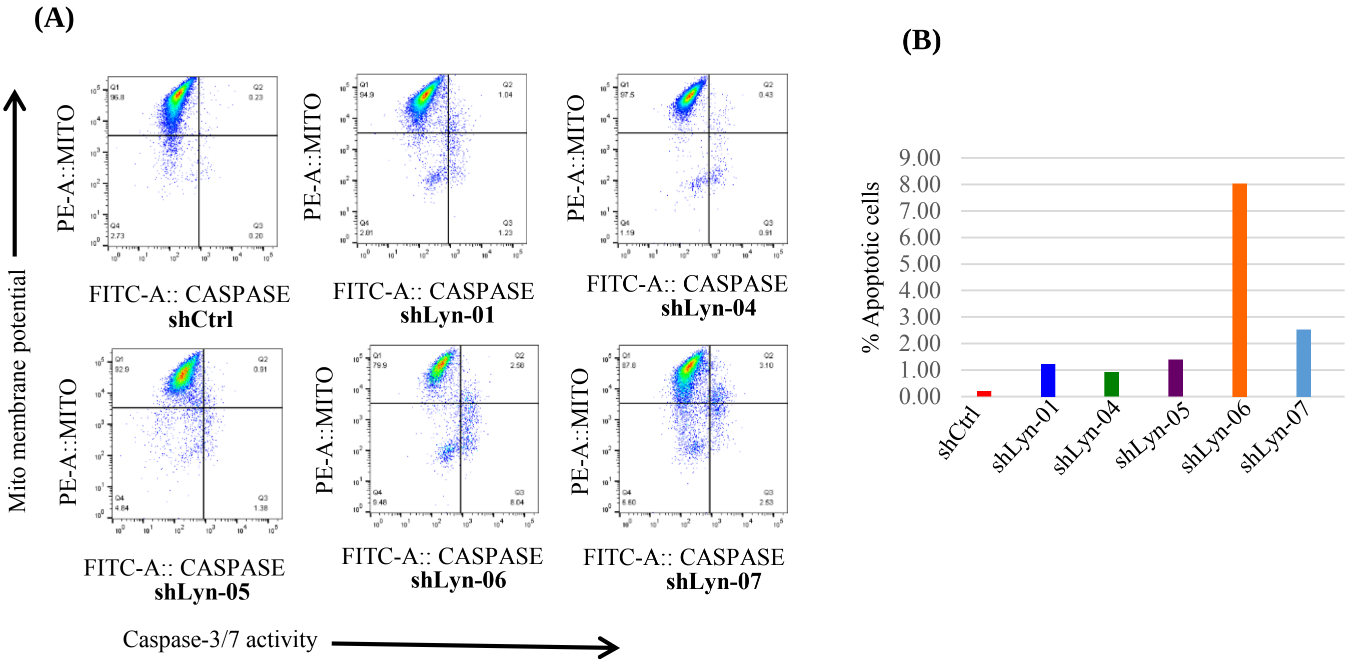

S4 Fig. Lyn knockdown in Myl-R cells lowered mitochondrial membrane potential and increased caspase-3/7 activity. (A) Lyn knockdown resulted in lower membrane potential and increased caspase-3/7 activity in MYL-R cells as determined by flow cytometry. Knockdown of Lyn was achieved by infecting MYL-R cells with lentiviral particles containing shRNA directed against Lyn. Fluorescence intensities for mitochondrial membrane potential and caspase-3/7 activity for Lyn knockdown MYL-R cells (shCtrl, shLyn-01, shLyn-04, shLyn-05, shLyn-06, and shLyn-07) were measured using the MitoCasp^{TM} dual sensor. (B) The most efficient anti-Lyn shRNA construct (shLyn-06) yielded the highest percent of apoptotic cells as determined by the fraction of cells in quadrant 3 (Q3).
