# Supplementary material for: BIRC6 mediates imatinib resistance independently of Mcl-1
Source: PLoS One. 2017 May 16;12(5):e0177871. doi: 10.1371/journal.pone.0177871 (PMC5433768; doi:10.1371/journal.pone.0177871)
Supplement: S5 Fig — (A) CK2 inhibition substantially reduced BIRC6 protein. MYL-R cells were treated with CX-4945, a small molecule inhibitor of CK2, in a time-course manner and cells harvested after 24, 48, and 72 hours. Immunoblot analyses were performed to examine BIRC6 protein and activation level of a validated CK2 substrate (phospho-IF2β). (B) BIRC6 was immunoprecipitated from lysates of MYL-R cells. The supernatant and beads-only lanes showed no BIRC6 protein as determined by immunoblot analysis, and (C) CK2 co-immunoprecipitated with BIRC6. CK2α was present in the BIRC6 IP but not in the beads-only control. (D) Baseline CK2 activity is the same in both MYL and MYL-R cells. MYL and MYL-R cells were lysed and immunoblot analyses performed to determine the activity level of CK2 (phospho-CK2β) and the level of active CK2 substrate (phospho-EEF1D). The data showed that CK2 activity was the same in MYL and MYL-R cells. (PDF) [file pone.0177871.s005.pdf]

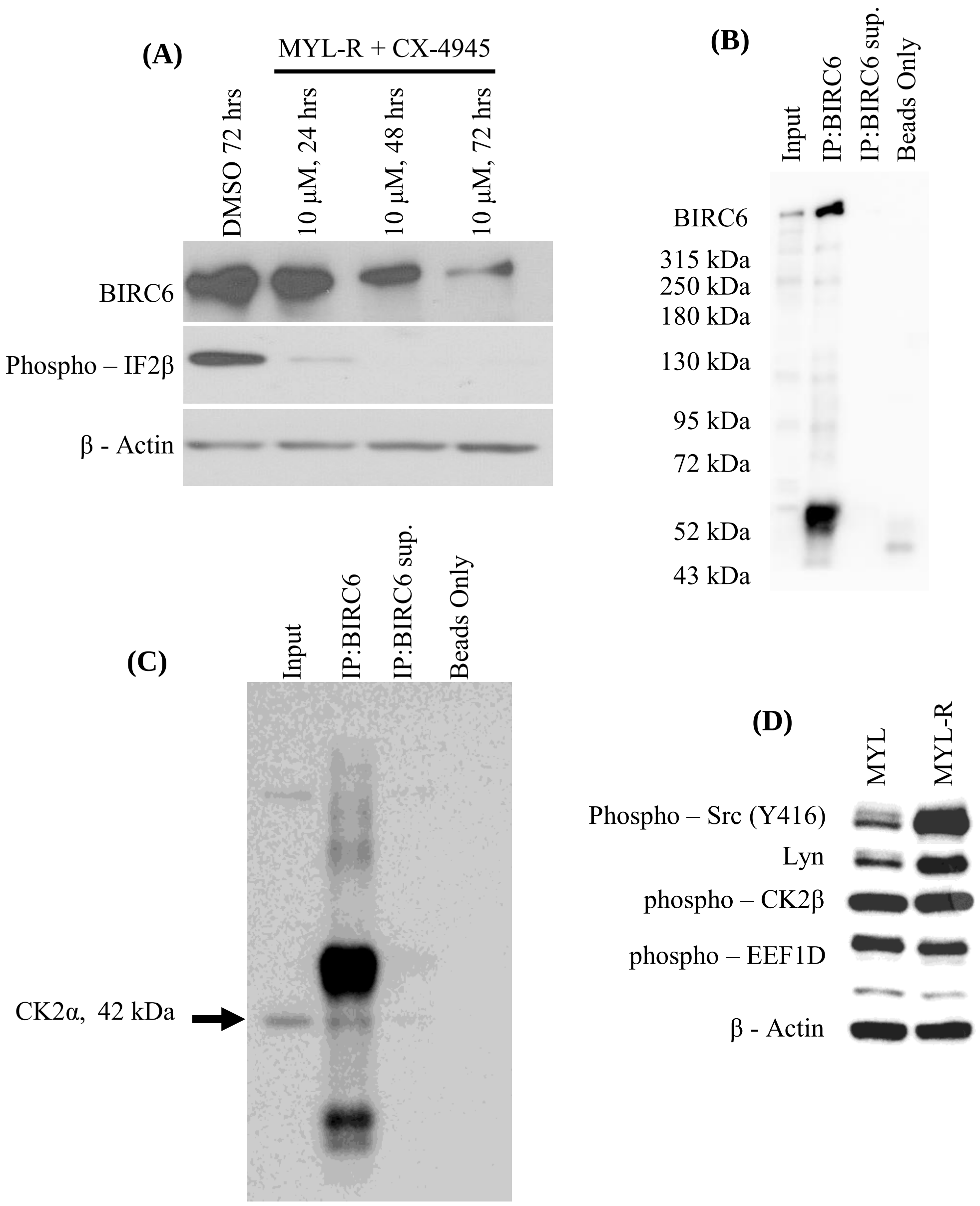

S5 Fig. (A) CK2 inhibition substantially reduced BIRC6 protein. MYL-R cells were treated with CX-4945, a small molecule inhibitor of CK2, in a time-course manner and cells harvested after 24, 48, and 72 hours. Immunoblot analyses were performed to examine BIRC6 protein and activation level of a validated CK2 substrate (phospho-IF2β). (B) BIRC6 was immunoprecipitated from lysates of MYL-R cells. The supernatant and beads-only lanes showed no BIRC6 protein as determined by immunoblot analysis, and (C) CK2 co-immunoprecipitated with BIRC6. CK2α was present in the BIRC6 IP but not in the beads-only control. (D) Baseline CK2 activity is the same in both MYL and MYL-R cells. MYL and MYL-R cells were lysed and immunoblot analyses performed to determine the activity level of CK2 (phospho-CK2β) and the level of active CK2 substrate (phospho-EEF1D). The data showed that CK2 activity was the same in MYL and MYL-R cells.
