# Supplementary material for: BIRC6 mediates imatinib resistance independently of Mcl-1
Source: PLoS One. 2017 May 16;12(5):e0177871. doi: 10.1371/journal.pone.0177871 (PMC5433768; doi:10.1371/journal.pone.0177871)
Supplement: S3 Fig — (A) Ponatinib more effectively suppressed Bcr-Abl and Lyn signaling, and BIRC6 protein than imatinib. MYL-R cells were treated with increasing concentrations of imatinib or 10 nM ponatinib or 0.1% DMSO for 24 hours and immunoblot analyses performed to examine the effects on BIRC6, phospho-Bcr-Abl, and Bcr-Abl/Lyn substrate, Crkl. (B) BIRC6 knockdown in MYL-R cells did not affect either phospho-Crkl or total Crkl. By contrast, BIRC6 knockdown caused substantial decrease in both phospho-Bcr-Abl and total Abl. (C) MYL-R cells had delayed activation of caspase-3/7 in response to imatinib treatment relative to MYL cells. MYL and MYL-R cells were treated with 1 μM imatinib in a time-course manner: 0, 6, 12, 24, 48 and 72 hours. Treatment was scheduled so that all cells were harvested at the 72-hr time-point. Caspase-3/7 activity was measured for each condition using a fluorogenic assay. MYL cells showed a two-fold higher basal caspase-3/7 activity relative to MYL-R cells. (PDF) [file pone.0177871.s003.pdf]

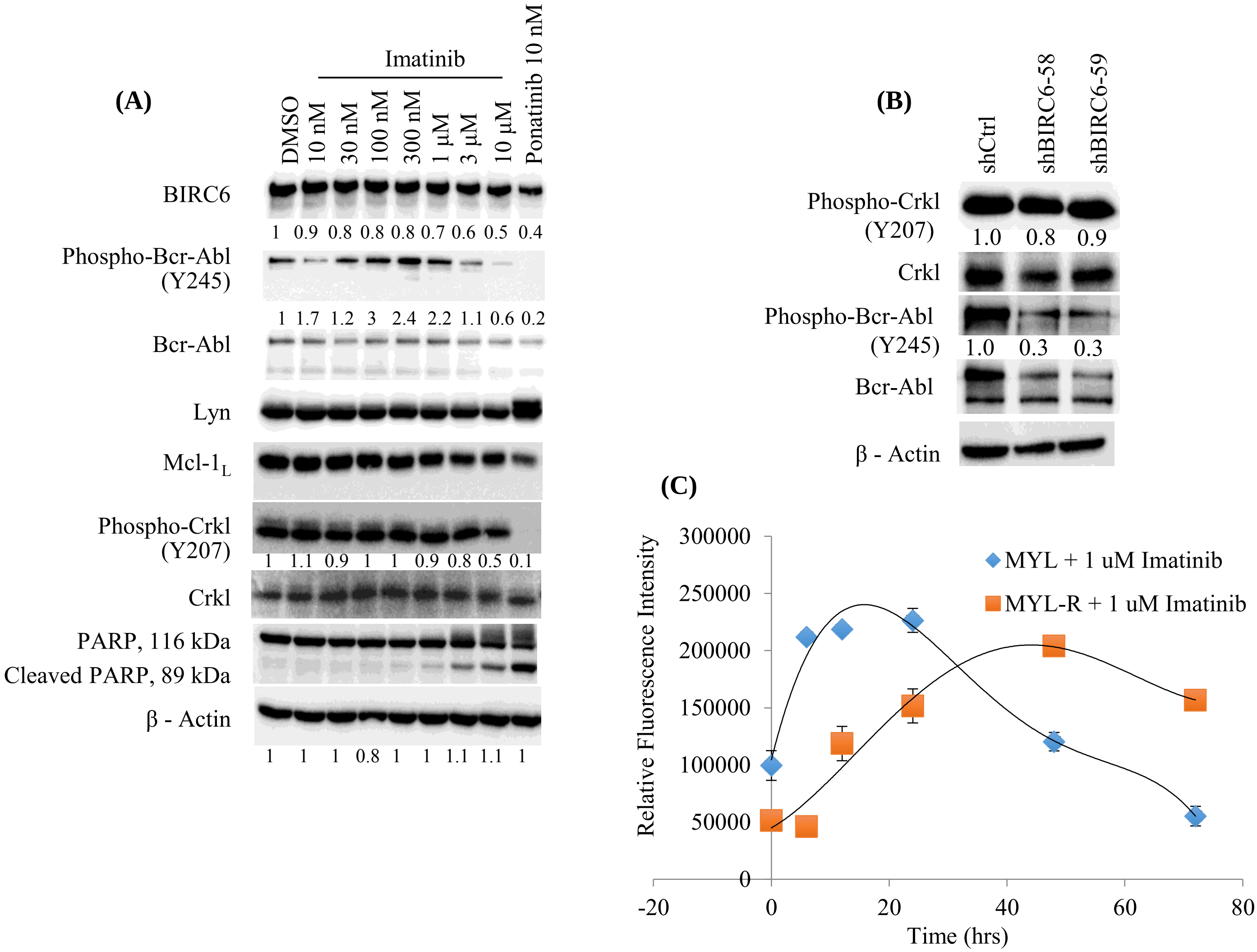

S3 Fig. (A) Ponatinib more effectively suppressed Bcr-Abl and Lyn signaling, and BIRC6 protein than imatinib. MYL-R cells were treated with increasing concentrations of imatinib or 10 nM ponatinib or 0.1% DMSO for 24 hours and immunoblot analyses performed to examine the effects on BIRC6, phospho-Bcr-Abl, and Bcr-Abl/Lyn substrate, Crkl. (B) BIRC6 knockdown in MYL-R cells did not affect either phospho-Crkl or total Crkl. By contrast, BIRC6 knockdown caused substantial decrease in both phospho-Bcr-Abl and total Abl. (C) MYL-R cells had delayed activation of caspase-3/7 in response to imatinib treatment relative to MYL cells. MYL and MYL-R cells were treated with 1 µM imatinib in a time-course manner: 0, 6, 12, 24, 48 and 72 hours. Treatment was scheduled so that all cells were harvested at the 72-hr time-point. Caspase-3/7 activity was measured for each condition using a fluorogenic assay. MYL cells showed a two-fold higher basal caspase-3/7 activity relative to MYL-R cells.
